# Incorporating environmental costs into long-term open-pit mine planning for sustainable resource optimization

**DOI:** 10.1038/s41598-026-51396-z

**Published:** 2026-05-21

**Authors:** Ali Salehi, Morteza Osanloo, Sajjad Afraei

**Affiliations:** Department of Mining Engineering, Amirkabir University of Technology, Tehran, Iran

**Keywords:** Open pit mining, Sustainable mining, Environmental assessment, Production planning, Engineering, Environmental sciences, Environmental social sciences

## Abstract

Long-term production planning in open-pit mines should be conducted in a manner that satisfies current societal demand for mineral resources while maintaining an appropriate balance with environmental protection. Achieving such a balance requires that environmental considerations be systematically incorporated into mine design and production planning processes. Given the absence of a comprehensive framework for quantifying environmental costs in production planning, this study identifies the various categories of these costs and proposes practical methods for their quantification. Accordingly, a Mixed-Integer Linear Programming (MILP) model is developed in which all environmental costs are directly and consistently integrated into the objective function based on ore and waste rock types. The proposed model enables the coherent integration of all environmental costs associated with open-pit mining into the production planning process and can be fully implemented within a three-dimensional block model framework. The results show that although the ultimate pit limit and production schedule derived from the proposed model lead to a lower apparent NPV, they offer a more realistic representation of actual mining conditions. A comparison of the evaluated scenarios indicates that directly incorporating environmental costs into the objective function of the proposed MILP model increases the total mine NPV in the case study by approximately 14.87% compared with a scenario that block model is designed as in conventional models in which environmental costs are excluded from the objective function and the total mine NPV is calculated including the related environmental costs. This result highlights that integrating environmental costs at the optimization stage supports more realistic and sustainability-oriented decision-making. In addition, the cost distribution analysis demonstrates that acid mine drainage (AMD) control costs and greenhouse gas (GHG) emission costs are the most influential environmental cost components affecting the project’s economic performance.

## Introduction

Recently, increasing attention has been directed toward environmental and sustainability issues in mining, accompanied by efforts to mitigate the adverse impacts of this industry on the environment and society. The adoption of advanced technologies and sustainable approaches in mining provides an opportunity to reduce these impacts and to achieve a balance between economic needs and environmental preservation. Mining, as a fundamental yet complex industry, requires careful and responsible management in order to both contribute to economic growth and safeguard natural resources for future generations. To achieve the objectives of sustainable development in the mining sector, a balance must be maintained between economic progress, environmental protection, and social well-being. The mining industry plays a vital role in supplying essential raw materials for both domestic and global industries. This, in turn, contributes to infrastructure development, job creation, and income generation for local communities. However, ensuring sustainability in this sector necessitates the careful management of environmental and social impacts so that economic development does not come at the expense of environmental degradation or the decline of quality of life for local populations^1^.

One of the most critical aspects of sustainable management in open-pit mining is the determination of the ultimate pit limit and long-term production planning. These processes play a fundamental role in enhancing productivity, reducing operational costs, and defining the most effective strategies for mineral extraction. The determination of the ultimate pit limit is carried out using various methods that consider multiple criteria, including mining costs, mineral prices, processing and refining expenses, as well as environmental factors. Accurate delineation of this boundary is of great importance, as it has a direct impact on both the profitability and the life of the mine. Long-term production planning in open-pit mines is also undertaken to ensure the efficient utilization of resources and the fulfillment of the mine’s financial and operational objectives. This planning involves defining the extraction sequence, allocating resources, and managing different mining processes in order to minimize costs and maximize efficiency throughout the mine’s life cycle^2^.

Given the importance of sustainable mining, the design and long-term production planning of open-pit mines must be conducted with consideration of the associated environmental costs. At present, long-term mine production planning is generally performed as a single-objective process, focusing solely on mining and processing costs, while the environmental costs and consequences of mining activities are often overlooked. Variations in production planning can lead to changes in the mined land area, waste dump sites, tailings dams, and the amount of GHG emissions. To integrate environmental costs into long-term production planning, it is necessary first to quantify these costs per ton of rock and then incorporate them into the production planning model. Accordingly, the aim of this study is to identify the significant environmental impacts related to ultimate pit limit determination and production planning, and to quantify their costs on a per-ton basis. Furthermore, a MILP model has been developed in this study for long-term production planning in iron ore mines, with various categories of environmental costs incorporated into the model. This approach offers an effective framework for embedding environmental considerations into strategic decision-making in the mining sector.

## Literature review

Although environmental assessment of mining projects is mandated by law in many countries, such assessments are typically conducted independently from the mine design process^3^. Production planning optimization, which constitutes a critical stage in mine design, has a significant impact on both economic and environmental outcomes. Key elements of this planning include the production rate, extraction sequence, overall pit slop and mine life. A review of previous studies on production planning indicates that the objective function of optimization has consistently focused on maximizing economic benefits, while environmental considerations associated with such planning have been largely overlooked^4^. Moreover, in most prior research, the optimal ultimate pit limit of an open-pit mine was first determined with the objective of maximizing mining profit, followed by long-term production planning within the same boundary aimed at maximizing the NPV. However, the determination of the ultimate pit limit and production planning should be undertaken in an integrated and simultaneous manner, with the common objective of maximizing NPV.

In recent years, research on determining the optimal ultimate pit limit and sustainable production planning can be categorized into four main areas:

- Solution methodologies: application of heuristic and metaheuristic approaches
- Problem size reduction and clustering
- Integration of uncertainties
- Incorporation of environmental costs

Since long-term production planning in open-pit mines is an NP-hard problem and cannot be solved using exact mathematical methods at large scales within reasonable time, many researchers have shifted toward heuristic and metaheuristic approaches to address this challenge^5–10^. Early attempts include Dagdelen^11^^,^ who applied Lagrangian relaxation with subgradient optimization to solve the long-term production planning (LTPP) problem, followed by Tolwinski and Underwood^12^^,^ who combined dynamic programming, stochastic optimization, artificial intelligence, and heuristic rules. Denby and Schofield^13^ employed genetic algorithms as a metaheuristic approach for open-pit design and production scheduling, aiming to maximize net present value while disregarding economic and social uncertainties. Similarly, Djilani^14^ proposed a hybrid method integrating Matron’s parametric approach with Whittle’s algorithm. Achireko and Frimpong^15^ employed artificial neural networks (ANN) for production scheduling and open-pit limit optimization. Latorre and Golosinski^16^ combined dynamic programming with a heuristic method for pit optimization under specific economic and discounting conditions, while Nanjari and Golosinski^17^ extended this algorithm to incorporate sequencing and waste stripping considerations. Metaheuristic approaches have continued to receive attention in mine planning research. For example, Sattarvand,J.(2009)^18^ applied ant colony optimization to production scheduling. Moosavi et al^.^^19^ combined genetic algorithms (GA) with Lagrangian relaxation to solve the large-scale long-term production scheduling problem. Lamghari and Dimitrakopoulos^20^ introduced a hyper-heuristic combining reinforcement learning and tabu search to address complex stochastic mine planning problems, demonstrating superior performance over existing metaheuristics while Loor and Morales^21^ developed an AI-based framework combining genetic algorithms and k-means clustering for block sequencing. Jélvez et al^.^^22^ proposed a hybrid heuristic for long-term production scheduling that integrates rolling-horizon decomposition with block pre-selection, enabling fast generation of near-optimal solutions. Rahmanpour et al^.^^23^^,^ who introduced a heuristic to reduce the number of binary variables in large-scale models,and Nancel-Penard et al^.^^24^^,^ who developed the recursive time aggregation–disaggregation (RAD) heuristic for complex multi-period precedence-constrained problems. Genetic algorithms remain widely applied, as demonstrated by Amponsah et al^.^^25^, who proposed a GA-based framework for mine production scheduling^26^. Finally, these studies demonstrate a gradual shift from classical optimization methods toward more advanced heuristic and metaheuristic techniques, enabling the solution of larger and more complex open-pit mine planning problems. Although these methods do not guarantee absolute optimality and their solution quality is highly dependent on parameter tuning, in practice they have been capable of generating near-optimal solutions within acceptable computational times^27^.

In recent years, another group of researchers has focused on reducing the problem scale in order to facilitate and accelerate the solution process. This approach is typically implemented through zoning of the ore block model or dividing it into several mining pushbacks, after which long-term production planning is carried out separately for each section^28–33^. In addition, some studies have employed clustering algorithms such as K-Means or hierarchical clustering to group blocks based on their intrinsic and operational parameters (e.g., grade, rock type, and spatial location)^23,34–39^. These methods reduce the number of variables and decision parameters, thereby significantly decreasing computational time,however, this may come at the expense of reduced accuracy due to the loss of block-level detail.

On the other hand, given the existence of economic and geological uncertainties that directly affect production planning and the economic value of any mining project, some researchers have attempted to incorporate these uncertainties into long-term production planning models^40–45^. Most studies have focused on two main types of uncertainties: economic uncertainties, which directly affect the economic value of the project and strategic decision-making—such as price volatility, fluctuations in operating costs, and discount rates^46–49^,and geological uncertainties, which primarily influence the estimation of reserve tonnage and grade as well as operational constraints, including grade variability and reserve-tonnage variability^5,50–56^.

Mining activities, particularly in open-pit operations, generate significant environmental impacts. On the one hand, large areas of land are occupied and disturbed for the pit, waste dumps, tailings dams, and related infrastructure^57^,on the other hand, the waste materials generated during extraction may cause water and soil contamination, acid mine drainage, ecosystem degradation, and alterations to the natural landscape^58,59^. To mitigate these impacts, certain reclamation methods are applied at the end of mining operations,however, such measures are generally implemented only after extraction has ceased and are unable to fully eliminate the consequences throughout the mine’s life cycle^60,61^. Therefore, it is essential that the design and planning of open-pit mines be carried out in a way that intrinsically minimizes environmental impacts within the decision-making process^4^.

Environmental studies in open-pit mines can be classified into four main categories:

- Land reclamation
- Mitigation of water and soil pollution caused by acid mine drainage and tailings leakage
- Reduction of air pollutant emissions such as CO₂, SO₂, NOx, and particulate matter (dust)
- Life Cycle Assessment (LCA) to evaluate environmental impacts throughout the entire extraction period

Despite the importance of these areas, production planning optimization in most studies has been conducted solely based on maximizing the NPV, without incorporating environmental costs into the decision-making model. However, production planning directly influences annual volumes of ore and waste extraction, the amount of greenhouse gas emissions, the extent of land disturbance, and disruptions to ecosystems^62^.

The main challenge in this area lies in the comprehensive quantification of environmental costs. Many studies have considered these costs solely as equivalent to land reclamation expenses, while overlooking other consequences such as water and air pollution, habitat loss, and soil erosion. The reasons for this shortcoming include the difficulty of collecting precise local data, the absence of unified international standards, and the requirement for long-term datasets^63^. Moreover, different ultimate pit limit designs can lead to significant variations in ore and waste extraction volumes, energy consumption, the location and size of waste dumps, and the level of pollutant emissions^63–65^ Over time, these differences alter both the distribution and magnitude of environmental costs.

**Fig. 1:**
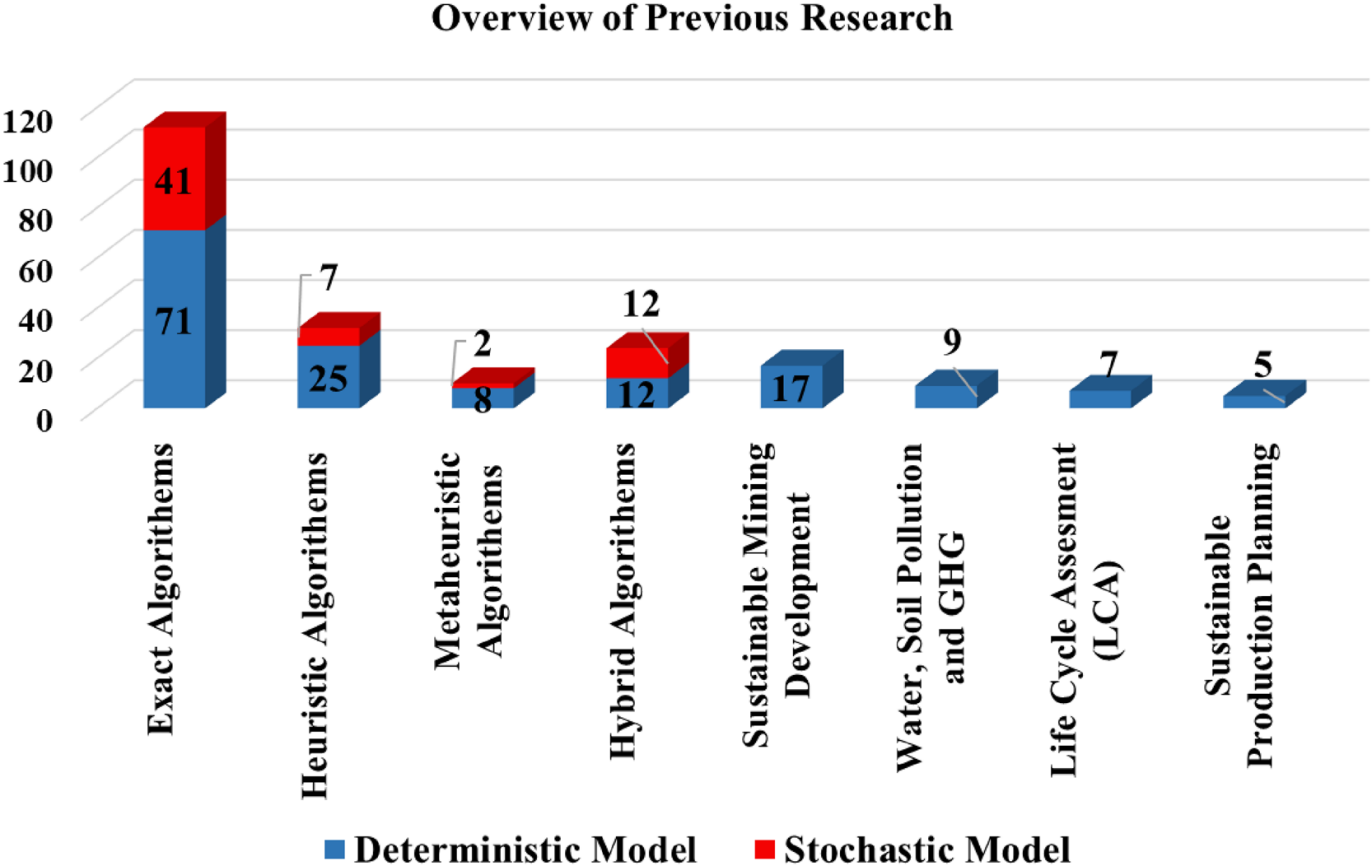
Thematic classification of previous studies (1965–2025).

A review of previous research reveals that only a limited number of studies have explicitly addressed the quantification of environmental costs in long-term production planning. Even in those instances, attention has typically been restricted to a subset of such costs rather than encompassing their full spectrum. For example, Muñoz et al^66^ were among the first to propose formulations for quantifying selected environmental costs, although their study considered only a limited subset of those relevant to long-term mine planning. Building on this, Xu et al^10^ introduced a heuristic method for determining the ultimate pit limit while incorporating certain environmental costs alongside economic and social factors. More recently, Mirzehi and Afrapoli^67^ developed an innovative framework for integrating greenhouse gas emission costs into the long-term production planning process of open-pit mines, contributing to the advancement of sustainable and green mining practices. To date, however, no comprehensive study has been undertaken that systematically identifies, quantifies, and fully integrates all relevant environmental costs into a unified mathematical framework for ultimate pit limit determination and production planning. Figure 1 presents the distribution of studies across the various thematic areas considered in the literature.

The existing literature indicates that no study to date has comprehensively identified, quantified, and incorporated all environmental costs in an integrated manner into a single decision-making model for ultimate pit limit determination and long-term production planning in open-pit mines. This research extends beyond previous studies by identifying all categories of environmental costs and developing quantitative formulations for them, as earlier works either omitted these costs entirely or addressed only a subset. To this end, a MILP model has been developed that directly incorporates all quantified environmental costs into the production planning process. The model has been applied to a hypothetical three-dimensional block model, whereas most prior studies were limited to two-dimensional models. Furthermore, this approach simultaneously determines the optimal ultimate pit limit and production planning within a single framework, maximizing NPV while fully accounting for environmental costs.

## Materials and methods

In this study, a comprehensive approach was initially examined to quantify the environmental costs resulting from open-pit mining activities. Subsequently, these costs were considered for integration into the long-term production planning process of open-pit mines within the framework of sustainable development. To this end, the environmental impacts and components influencing production planning were first identified, and then a method was proposed to calculate the environmental costs per ton of rock (including both ore and waste rock). In the next step, to incorporate environmental considerations into long-term production planning, a mathematical model was developed. This model is formulated as a MILP problem and has been specifically designed for integrated planning in iron ore open-pit mines.

In this study, various cost components influencing the long-term production planning of iron ore open-pit mines were identified and incorporated into the model, as follows:

- **Carbon emission costs resulting from fuel and energy consumption**, including emissions from extraction operations (drilling, blasting, loading, hauling, and ancillary) and mineral processing (crushing, grinding, and magnetic separation).
- **Acid Mine Drainage (AMD) costs**, arising from the generation of acidic drainage due to the presence of iron sulfides in waste dumps of iron ore open-pit mines.
- **Lost value of direct ecosystem services**, referring to the cost of land acquisition for mining activities. In this study, the areas occupied by the mine pit, waste dump, and tailings dam are considered as mining lands.
- **Land reclamation costs**, associated with restoring the mined lands, including the mine pit, waste dump, and tailings dam.
- **Lost value of indirect ecosystem services**, encompassing the value of air purification, oxygen production, water resource conservation, soil erosion prevention, and nutrient cycling.

**Fig. 2:**
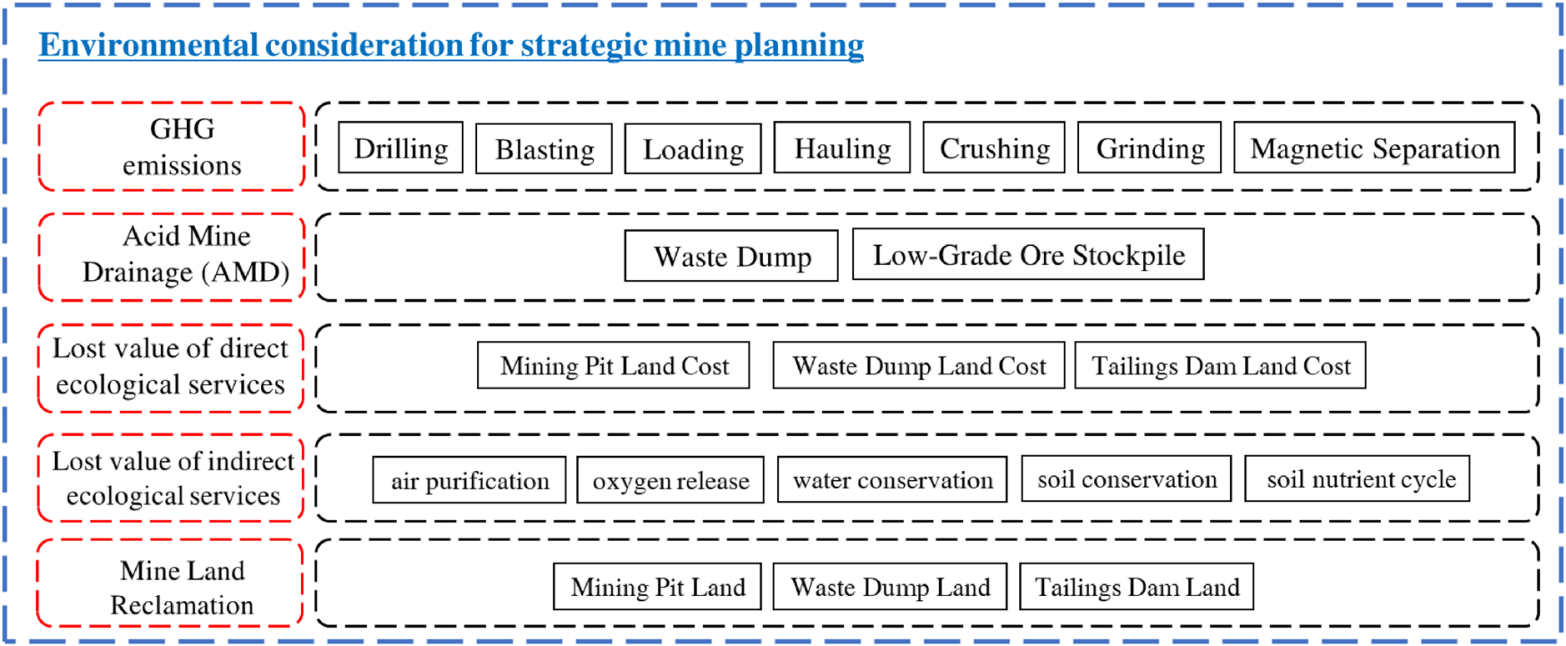
Environmental cost components considered in strategic mine planning.

Figure 2 presents the various environmental costs affecting strategic planning of open-pit iron ore mines, classified and detailed by their respective components.

The mathematical relationships used for the quantification of environmental costs were derived from both previously published formulations and new equations developed in this study. A complete list of all equations together with their corresponding sources is provided in Appendix A.

**Table:** 

**Symbol**	**Description**	**Unit**	**Symbol**	**Description**	**Unit**
${GHG}_{Drilling}$	${CO}_{2eq}$ emissions from drilling	kg ${CO}_{2eq}$/ton	${C}_{GHG}^{o}$	Carbon cost associated with ore	USD/ton
$GH{G}_{Blast}$	Greenhouse gas emissions from blasting operations	kg CO₂-eq/ton	${C}_{AMD}$	AMD control cost	USD/ton
${GHG}_{loading}$	Greenhouse gas emissions from loading operations	kg CO₂-eq/ton	${C}_{p}^{\mathrm{d}}$	Land disturbance cost of mined rock	USD/ton
${GHG}_{Hauling}$	Greenhouse gas emissions from haulage operation	kg CO₂-eq/ton	${C}_{w}^{\mathrm{d}}$	Land disturbance cost (direct ecological service cost) per ton of waste	USD/ton
${GHG}_{Ancillary}$	CO₂-equivalent emissions from ancillary activities	kg CO₂-eq/ton	${C}_{t}^{\mathrm{d}}$	Tailings dam Land disturbance cost (direct ecological service cost)	USD/ton
${GHG}_{cr}$	CO₂-eq emissions associated with Crushing	kg CO₂-eq/ton	${C}_{p}^{r}$	Reclamation cost of pit-disturbed land	USD/ton
${GHG}_{gr}$	CO₂-eq emissions associated with Grinding	kg CO₂-eq/ton	${C}_{w}^{r}$	Reclamation cost of waste dump land	USD/ton
${GHG}_{Mag}$	Greenhouse gas emissions from magnetic separation	kg CO₂-eq/ton	${C}_{t}^{r}$	Reclamation cost of tailings dam land	USD/ton
${GHG}_{W}$	CO₂-equivalent emissions per ton of waste rock	kg CO₂-eq/ton	${C}^{EE}$	Total Lost Value of Indirect Ecological Services	USD/ton
${GHG}_{O}$	CO₂-equivalent emissions per ton of ore	kg CO₂-eq/ton	${Q}_{w}$	Total waste from the maximum resource pit	ton
${T}_{GHG}$	Carbon tax	USD/ton CO₂-eq	${\mathrm{Q}}_{O}$	Mass of Ore	ton
${C}_{GHG}^{w}$	Carbon cost associated with waste rock	USD/ton	${Q}_{t}$	Mass of tailings	ton

*The main parameters and their definitions are summarized in this Table, while the full nomenclature is provided in the Appendix B.

### Carbon emission costs from energy and explosives consumption

**Fig. 3:**
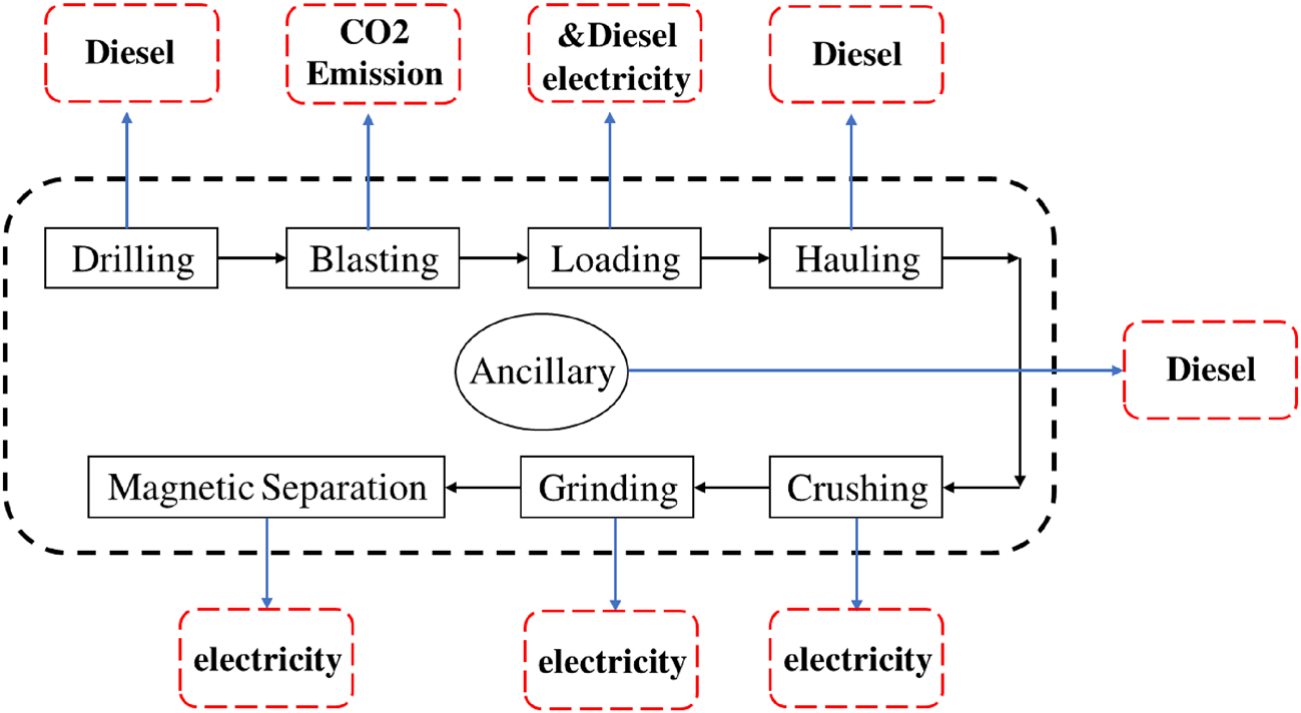
System framework considered in this study and the type of energy source used in each stage.

To quantify the GHG emission costs resulting from fuel and energy consumption in open-pit iron ore mining operations, the processes involving fuel and energy use are first identified. For each stage, the type of energy source (fuel or electricity) and the required consumption levels are determined. Based on the consumption of energy and explosives in each stage and the corresponding emission factors, the amount of GHG emissions per ton of rock is calculated. Figure 3 illustrates the system framework considered in this study and the type of energy source used in each process stage.

In general, energy and fuel consumption are considered in two main stages of iron ore mining: extraction and mineral processing. According to Fig. 3, iron ore extraction consists of four operations: drilling, blasting, loading, and hauling, along with ancillary operations. The mineral processing stage includes three processes: crushing, grinding, and magnetic separation, for which the energy and fuel consumption are calculated separately. The carbon emission costs in the extraction stage are allocated to both ore and waste rock, whereas the GHG emission costs resulting from energy consumption in the processing stage are only assigned to the ore.

#### Extraction stage

To determine the carbon emission costs in the extraction stage, four main operations—drilling, blasting, loading, and hauling as well as an ancillary operation are considered. In each operation, energy consumption and GHG emissions are calculated separately. The GHG emission costs resulting from fuel and energy consumption in the extraction stage are calculated for both ore and waste rock.

##### Energy consumption in drilling operations

The calculation of energy consumption in drilling operations for open-pit mining can be performed using three methods. The first method is based on the specific drilling energy characteristics; the second method relies on the operational power of the drilling equipment used; and the third method estimates fuel consumption based on the drilling rate in open-pit mines.

- Calculation of GHG emissions based on specific drilling energy

In this method, knowledge of the specific drilling energy for the rock type is required, and the energy consumption as well as the GHG emissions resulting from energy use in this operation are determined using Equations (1) and (2) ref^66^.

1 $${GHG}_{Drilling}=\left(\frac{A\cdot L\cdot N\cdot {E}_{V}}{{\upeta}_{Driller}\cdot {m}_{B}}\right)\times \zeta$$

2 $${GHG}_{Drilling}=\left(\frac{{E}_{V}}{{\upeta}_{Driller}\cdot {\rho}_{rock}}\right)\times \zeta$$

- Calculation of GHG emissions based on operational parameters of the drilling machine

In this method, the energy consumption for drilling operations can be calculated using operational parameters of drilling in the mine, such as the power of the drilling machine and the drilling time per blast hole. Equation (3) presents the calculation of energy consumption in drilling operations based on these operational parameters.

3 $${GHG}_{Drilling}=\left(\frac{P\times N\times {t}_{hole}\times 3.6}{{\upeta}_{Driller}\times {m}_{B}}\right)\times \zeta$$

- Calculation of GHG emissions based on drilling rate and diesel consumption

Equation (4) presents the calculation of GHG emissions based on the drilling rate and diesel consumption.

4 $${GHG}_{drillig}={R}_{d}\times \frac{{M}_{d}\times BF}{{P}_{r}}\times \delta$$

##### GHG emissions in blasting operations

In blasting operations, GHG emissions can be directly calculated based on the amount of explosive consumed and using available emission factors. For calculating GHG emissions from blasting, the quantity of gases produced per unit mass of explosive can be derived from its ideal chemical reaction. In the case of ANFO, the proportions of ammonium nitrate (AN) and fuel oil (FO) are balanced, with neither excess nor deficiency of oxygen in the explosive reaction. For every 254 kg of ANFO used, 44 kg of CO_2_​ is produced^66^. Subsequently, GHG emissions from blasting per ton of rock are calculated using Equation (5). When ANFO is used for dry areas and emulsion explosives are applied in wet blocks, GHG emissions from blasting can be calculated using Equation (6).

5 $$GH{G}_{\mathrm{Blast}}={Q}_{E}\times {\mathrm{LF}}$$

6 $$GH{G}_{Blast}=LF\times \left(\left({R}_{ANFO}\times {Q}_{A}\right)+\left({R}_{emulsion}\times {Q}_{E}\right)\right)\times {10}^{-3}$$

##### Energy consumption and GHG emissions in loading operations

Loading systems in open-pit mines are typically a combination of diesel and electric loading equipment. Therefore, to calculate GHG emissions in this operation, Equations (7) and (8) can be used, which determine, respectively, the energy consumption and the amount of diesel fuel consumed during loading operations.

7 $${GHG}_{loading}=\left(\frac{{P}_{L}\times T}{{\upeta}_{Loader}\times {m}_{Truck}}\right)\times \zeta$$

8 $${GHG}_{loading}={D}_{l}\times \delta$$

##### Energy consumption and GHG emissions in haulage operations

Haulage refers to the process of transporting extracted materials from the mine pit to destinations such as the crusher, waste dump, or low-grade ore stockpile. The energy consumption for moving each ton of rock, as well as the amount of diesel fuel consumed, varies depending on factors such as mining depth, the location of destinations, and the type of haulage system used. Equation (9) presents the calculation of the energy required for hauling ore and waste rock from the mine pit to various destinations. GHG emissions are calculated based on the required energy and the corresponding emission factors. Equations (10) and (11) are also used to determine the amount of diesel fuel consumed in haulage operations using diesel-powered equipment in open-pit mines, and to calculate the resulting GHG emissions based on the relevant emission factors.

9 $${GHG}_{Hauling}=\left(\frac{9.81\times S\times \left({m}_{Truck}\times i\mathrm{\%}+\left({R}_{s}\mathrm{\%}+{R}_{t}\mathrm{\%}\right)\times \left(2\times {M}_{Truck}-{m}_{Truck}\right)\right)}{{m}_{Truck}}\right)\times \zeta$$

10 $${GHG}_{Hauling}=\left(\frac{{T}_{cycle}\cdot FCR}{60\cdot {m}_{Truck}\cdot EF}\right)\times \delta$$

11 $${T}_{cycle}=\left(\frac{D}{{V}_{loaded}}+\frac{D}{{V}_{empty}}\right)\cdot 60+{T}_{load}+{T}_{dump}+{T}_{wait}$$

##### Energy consumption and GHG emissions in ancillary activities

The ancillary fleet includes loaders, graders, bulldozers, fuel trucks, and excavators with a capacity of less than 300 tons (secondary loading units). These machines are used for tasks such as^67^:

- bench preparation,
- maintenance of haul roads,
- reconstruction of temporary stockpiles,
- reclamation of waste dumps, and other ancillary activities.

The CO₂-equivalent emissions resulting from diesel consumption in these ancillary activities are calculated using Equation (12). In this equation, diesel consumption in ancillary activities is modeled per ton of material extracted.

12 $${GHG}_{Ancillary}={D}_{a}\times \delta$$

#### Processing stage

In the processing stage of iron ore, three main operations are considered: crushing, grinding, and magnetic separation. The carbon emission costs resulting from energy consumption in these processes are calculated only for the ore.

##### Energy consumption in crushing

The comminution stage, in which solid materials are reduced from an average size to a smaller size, is carried out through crushing processes. The CO₂-equivalent emissions associated with crushing one ton of ore can be calculated using Bond’s Law and Equation (13).

13 $${GHG}_{cr}=3.6\times 10\times {W}^{cr}\times \left(\frac{1}{\sqrt{{P}_{80}^{cr}}}-\frac{1}{\sqrt{{F}_{80}^{cr}}}\right)\times \zeta$$

##### Energy consumption in grinding

Grinding is the second stage of the comminution process, during which ore particles are reduced to finer sizes to achieve mineral liberation. Due to its high energy consumption, this process has a significant contribution to greenhouse gas emissions; therefore, estimating its environmental impacts using Bond’s Law and emission factors is of particular importance. The CO₂-equivalent emissions associated with grinding one ton of ore can be calculated using Bond’s Law and Equation (14).

14 $${GHG}_{gr}=3.6\times 10\times {W}^{gr}\times \left(\frac{1}{\sqrt{{P}_{80}^{gr}}}-\frac{1}{\sqrt{{F}_{80}^{gr}}}\right)\times \zeta$$

##### Energy consumption in magnetic separation

The greenhouse gas emissions generated during magnetic separation are calculated according to Equation (15), based on the power consumption of the magnetic separation equipment and the duration of the operation.

15 $${GHG}_{Mag}=\frac{{P}_{mag}\times {T}_{mag}\times 3.6}{{m}_{ore}}\times \zeta$$

#### Carbon pricing

GHG emissions are widely acknowledged as one of the most pressing global environmental issues^66^. In this research, the GHG emissions of each mining block are evaluated as a function of the energy utilized within that block. Furthermore, emissions generated from blasting are incorporated into the model and estimated based on an idealized chemical reaction. Thus, the overall GHG emissions linked to each block are represented as the sum of three components—electricity, fossil fuel, and explosives—combined in an aggregated equation.

To calculate the costs associated with carbon pricing in the extraction and processing stages, it is first necessary to identify the type of energy carrier or fuel used in each stage. Since the type of rock (waste or ore) and its final destination significantly affect GHG emissions, this study distinguishes between different rock types and their transportation destinations, such as delivery to the processing plant or to the waste dump. Finally, based on Equations (16) and (17), the GHG emissions resulting from energy consumption for each block are determined separately and proportionally to the amount of energy consumed.

16 $${GHG}_{W}={GHG}_{Drilling}+GH{G}_{Blast}+{GHG}_{loading}+{GHG}_{Hauling}+{GHG}_{Ancillary}$$

17 $$\begin{aligned}{GHG}_{O}& ={GHG}_{Drilling}+GH{G}_{Blast}+{GHG}_{loading}+{GHG}_{Hauling}\\ & \quad +{GHG}_{Ancillary}+{GHG}_{cr}+{GHG}_{gr}+{GHG}_{Mag}\end{aligned}$$

One of the effective mechanisms for controlling and reducing GHG emissions is the implementation of a carbon tax^66^. From a theoretical perspective, this type of tax can be considered a Pigouvian tax, defined based on the negative externalities resulting from GHG emissions. In modeling, this tax is incorporated as a new component into the objective function and is directly related to the amount of emissions generated from the extraction of each mining block. As a result, the extraction-related costs increase proportionally to the volume of GHG emissions produced, thereby influencing economic decision-making. The calculation of the carbon tax for ore and waste rock is performed using Equations (18) and (19).

18 $${C}_{GHG}^{w}=\frac{{T}_{GHG}\times {GHG}_{W}}{1000}$$

19 $${C}_{GHG}^{o}=\frac{{T}_{GHG}\times {GHG}_{O}}{1000}$$

### Cost of AMD generation

AMD is recognized as one of the most significant environmental challenges associated with mining activities worldwide. Its importance arises from its long-term impacts and widespread social, health, and environmental consequences across various regions. In fact, AMD has been identified as the most serious environmental issue facing the mining industry in North America^68^.

According to the Acuerdo Marco de Producción Limpia^69^ report, the occurrence of AMD requires the presence of four main factors:

- the presence of sulfide minerals alongside minerals with neutralizing properties,
- the presence of water,
- the presence of oxygen, and
- the presence of bacteria that catalyze oxidation reactions.

Among these four factors influencing AMD formation, only the presence of sulfide minerals depends on the geological characteristics of each mining block and is considered the primary and determining factor in this study. Given the potential presence of pyrite in iron ore deposits, assessing this factor is of particular importance. When pyrite exists in high-grade iron ore, such materials are transported to the processing plant and, due to processing controls, do not result in AMD generation. Conversely, if pyrite is identified in low-grade materials that are classified as waste and stored in the waste dump, the formation of AMD becomes possible. Accordingly, in this study, only the waste dump has been evaluated as a potential source of AMD generation.

To assess the potential for AMD generation in each mining block, two key indices are used: Net Neutralization Potential (NNP) and Neutralization Potential Ratio (NPR). These indices are calculated based on two fundamental parameters: Acidic Potential (AP) and Neutralizing Potential (NP), both expressed in kilograms of calcium carbonate equivalent (CaCO₃) per ton of sample. The NP and AP of each block are estimated using the commonly applied Acid-Base Accounting (ABA) method, which is conducted in accordance with standards provided by the United States Environmental Protection Agency^70^^,^^71^.

20 $$AM{D}_{Pot}=f\left(NNP,NPR\right)$$

21 $$NNP=NP-AP$$

22 $$NPR=\frac{NP}{AP}$$

23 $$NP = 83.3 \times {g}_{c} ;\hspace{1em}AP = 31.25 \times {g}_{s}$$

In this study, it is assumed that all sulfur present in a block is considered a potential source of acid generation, regardless of its mineral form. However, certain compounds, such as gypsum and barite, lack acid-generating capacity and, if present, may lead to an overestimation of the Acidic Potential (AP). Additionally, all carbon present is assumed to exist in the form of calcite (CaCO₃) and is considered the sole contributor to the Neutralizing Potential (NP). To incorporate the potential for AMD generation into the model, costs associated with controlling and mitigating the environmental impacts of waste blocks are included. These costs increase proportionally with the intensity of AMD potential. Various methods exist for reducing AMD in waste blocks, including encapsulation, freezing, blending with other materials, application of bactericides, and engineered barriers. The cost of implementing these methods depends on the geographical conditions of the dump site; notably, in humid or high-rainfall areas, the cost of AMD mitigation is generally higher than in arid regions. The intensity of AMD generation potential is determined based on the Table 1.

**Table 1:** AMD risk levels and corresponding cost factors^66,71^**.**

Condition	AMD Risk Level	AMD Control Cost Coefficient ($/ton)
NNP < –20 or (–20 ≤ NNP ≤ +20 and NPR < 1)	High	$${\gamma}_{1}$$
–20 ≤ NNP ≤ +20 and 1 ≤ NPR < 2.5	Medium	$${\gamma}_{2}$$
–20 ≤ NNP ≤ +20 and NPR ≥ 2.5	Low	$${\gamma}_{3}$$
NNP > +20	NAG	0

The cost of controlling AMD per ton of rock with acid-generating potential depends on the intensity of AMD generation potential, the selected control method, and the geographical conditions of the dump site, such as topography, rainfall, accessibility, site hydrology, and other factors. Equation (22) presents the calculation of AMD control costs.

24 $${C}_{AMD}={\gamma}_{i}\times \alpha \times {C}_{base}$$

### Lost value of direct ecological service cost

One of the ecological costs resulting from mining activities is land degradation. Land degradation can have varying impacts depending on the location of the mine and the type of vegetation and wildlife in the area. For this purpose, the lost value of direct ecological services related to the provision of biological products (such as timber or agricultural products) is considered an environmental cost resulting from mining operations^10^. The land acquisition cost represents the amount that a mining company must pay to landowners for the right to use the land. This cost reflects the direct lost value of degraded land (such as land productivity and related values), which can be represented by the price of land acquisition. This price may be determined using one of the following methods^10^:

- Price of comparable or identical land,
- Negotiated price with the landowner,
- Compensation amount determined by the government for land damage,
- Or calculated based on an income approach.

**Fig. 4:**
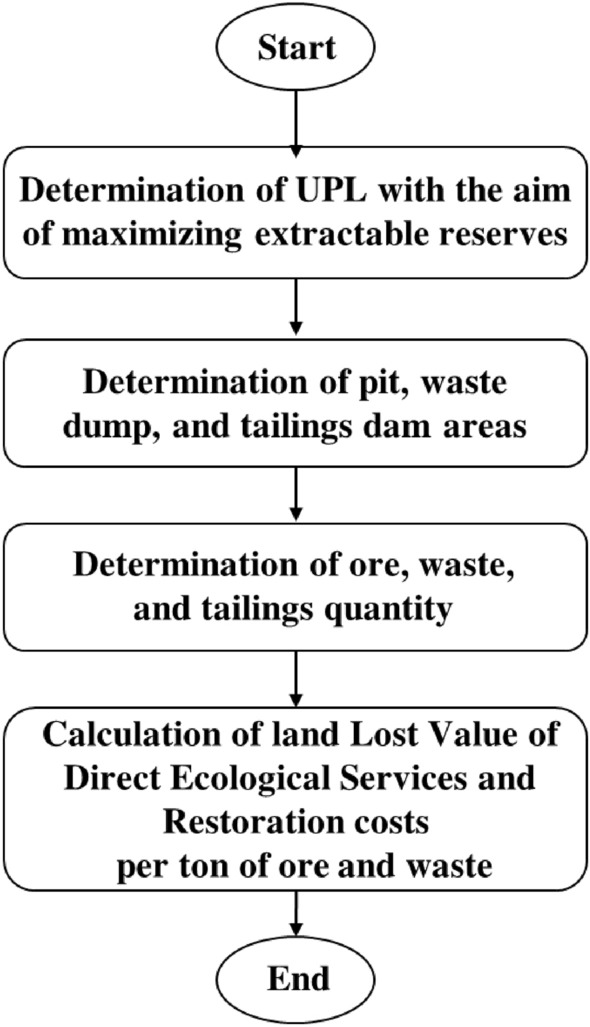
Flowchart for determining the unit-area cost of direct ecological services.

To calculate land degradation costs and land reclamation costs per ton of mined material, it is first necessary to define the final pit boundary based on the maximum extractable reserve and to determine the quantities of ore and waste rock contained within this pit, as well as the land area required for the waste dump and tailings dam. Subsequently, the cost of land degradation (direct ecological service cost) and the reclamation cost per ton of ore and waste rock are calculated. Figure 4 illustrates the steps for determining the ultimate pit and calculating the environmental cost per unit area.

To calculate the lost value of direct ecological services related to land degradation, the following two basic assumptions are considered:

Assumption 1) The environmental costs associated with the areas of the mine pit, waste dump, and tailings dam are taken into account.

Assumption 2) The cost of acquiring these lands is incurred at the beginning of the mine’s operational life.

Based on these assumptions, the cost of land degradation for the mine pit is calculated using Equation (25).

25 $${C}_{p}^{d}=\frac{{A}_{p}\times {c}_{g}}{{Q}_{tot}}$$

For the waste dump and tailings dam, the area of degraded land and the lost value of direct ecological services per ton are calculated using Equations (26) to (30).

26 $${A}_{w}=\frac{{Q}_{w}\cdot {f}_{w}}{{\uprho}_{w}\cdot {H}_{w}}\cdot {s}_{w}$$

27 $${C}_{w}^{d}=\frac{{A}_{w}\times {c}_{g}}{{Q}_{w}}$$

28 $${A}_{t}=\frac{{Q}_{t}}{{\uprho}_{t}\cdot {H}_{t}}\cdot {s}_{t}$$

29 $${C}_{t}^{d}=\frac{{A}_{t}\times {c}_{g}}{{Q}_{O}}$$

30 $${Q}_{t}=\left(1-\frac{{G}_{feed}\times R}{{G}_{conc}}\right)\times {Q}_{O}$$

### Reclamation costs

Mine reclamation costs are influenced by factors such as post-mining land use (PMLU) options, mining methods, the condition of the land after mining, and the characteristics of mine waste and tailings. Among these factors, the volume of earthworks required is the most significant, accounting for more than 70% of total mine reclamation costs. Generally, mine reclamation costs can be categorized into two groups: common costs and specific costs. Common costs relate to activities carried out to prepare the land for implementing various PMLU options and are consistent across similar reclamation projects, regardless of the selected PMLU option. These costs are mainly affected by the extent of earthworks required in each mine. In contrast, specific costs are associated with the implementation of the particular PMLU option chosen^60^. For more precise economic evaluations and better comparisons, calculating mine reclamation costs per ton of ore is essential.

Based on the reclamation plan, reclamation costs are incurred to return ecosystems damaged by mining activities to their original or expected conditions and also include maintenance costs after the completion of reclamation projects^72^. The reclamation cost for mined lands, including the areas of the mine pit, waste dump, and tailings dam, is calculated using Equations (31) to (33).

31 $${C}_{p}^{r}=\frac{{A}_{p}\times {c}_{r}}{{Q}_{tot}}$$

32 $${C}_{w}^{r}=\frac{{A}_{w}\times {c}_{r}}{{Q}_{w}}$$

33 $${C}_{t}^{r}=\frac{{A}_{t}\times {c}_{r}}{{Q}_{O}}$$

### Lost value of indirect ecological services

In addition to the lost value of direct ecological services provided by land, various land uses also offer a wide range of indirect services that contribute to human well-being. These services are referred to here as the lost value of indirect ecological services. However, human understanding of the functioning of ecological systems is still incomplete, and estimating the economic value of these services remains highly challenging or, in some cases, even impossible^4^.

In this study, to calculate the value of indirect ecological services in mining areas, five main services are considered: air purification, oxygen production, water resource conservation, soil erosion prevention, and nutrient cycling. These services vary in presence depending on the type of land use (such as forests, grasslands, and deserts), with different coefficients indicating the proportion contributed by each service in each land type. Accordingly, the economic value of these services is calculated separately for each land type, proportionate to their respective coefficients. The table 2 presents the coefficients corresponding to each land use type^73^^,^^74^.

**Table 2:** Magnitude and impact coefficients of indirect ecological services based on land use type *.

**Service**	**Land use type**		
	**Forest**	**Grassland**	**Desert**
Air purification	(None) 0	(Medium) 0.5	(High) 1
Oxygen production	(None) 0	(Medium) 0.5	(High) 1
Water conservation	(None) 0	(Medium) 0.5	(High) 1
Soil erosion control	(Low) 0.5	(High) 1	(High) 1
Nutrient cycling	(Very Low) = 0.2	(Medium) 0.5	(High) 1

*Developed in this study.

#### Air purification value

To calculate the economic value of the indirect air purification service, the ecosystem’s capacity to absorb or remove various pollutants such as CO₂, SO₂, NOₓ, particulate matter (dust), and to release beneficial compounds like phytoncides is first estimated. These quantities are then multiplied by the replacement cost or the economic value of removing these pollutants through artificial methods. The sum of these values represents the economic value of the ecosystem’s air purification service^4^. This approach helps express the impacts of land degradation on environmental costs in monetary terms. The air purification value for the degraded lands of the mine pit, waste dump, and tailings dam is calculated using Equation (34).

34 $${C}^{a}=\frac{\left({\sum}_{i=1}^{n}{Q}_{i}\times {C}_{i}\right)\times {\alpha}_{l}\times A}{Q}$$

i represents the different pollutants or compounds that the land can absorb, remove, or mitigate. In this study, these include:

- CO₂ → Carbon sequestration
- SO₂ → Absorption of acidic gaseous pollutants
- NOₓ → Absorption of nitrogen oxides
- Dust → Dust suppression
  $${\mathrm{i}}{\kern 1pt} { = }\,\left[ {{\mathrm{CO}}_{{2}} ,{\mathrm{SO}}_{{2}} ,{\mathrm{NO}}_{{\mathrm{X}}} ,{\mathrm{Dust}}} \right]$$

#### Oxygen production value

Oxygen production is one of the important indirect services provided by ecosystems, and its capacity varies depending on land use type. This capacity is usually calculated based on the Net Primary Production (NPP) of each ecosystem and the oxygen release coefficient. To estimate the economic value of oxygen production, the cost of producing the same amount of oxygen through industrial methods is considered. In this study, oxygen production across all land use types will be calculated in proportion to the coefficients representing the presence of indirect ecological services.

35 $${C}^{O}=\frac{\left({q}_{f}\times {\lambda}_{o}\times {c}_{o}\times {\alpha}_{l}\right)\times A}{Q}$$

#### Water conservation value

Water conservation is one of the important indirect services provided by ecosystems, and its capacity varies depending on land use type. The value of water conservation in ecosystems is calculated based on runoff reduction, average annual precipitation, and the proportion of effective rainfall contributing to runoff generation. In this method, the volume of water conserved in the ecosystem due to reduced runoff is first estimated and then multiplied by the cost of storing the same amount of water in artificial structures such as dams to determine its economic value. In this study, this value will be calculated for different land use types in proportion to the coefficients representing the presence of indirect ecological services.

36 $${C}^{h}=\frac{\left(10\times j\times k\times {r}_{h}\times {c}_{h}\times {\upalpha}_{l}\right)\times A}{Q}$$

#### Soil erosion control value

Soil conservation is one of the important indirect services provided by ecosystems, playing a significant role in reducing both water and wind erosion. This service prevents land degradation and the conversion of land into unusable areas. The economic value of soil conservation is typically estimated using the opportunity cost method, whereby the amount of soil preserved is equated to an area of agricultural land that would require replacement or reclamation if the soil were lost. In this study, the value of soil conservation will be calculated for different land use types in proportion to the coefficients representing the presence of indirect ecological services.

37 $${C}^{s}=\frac{\left(\frac{{S}_{p}\times\uprho \times h\times v}{\mathrm{10,000}}\times {\upalpha}_{l}\right)\times A}{Q}$$

#### Nutrient cycling value

Nutrient cycling is one of the important indirect services provided by ecosystems, leading to the production and retention of essential soil elements such as nitrogen (N), phosphorus (P), and potassium (K). The quantities of these nutrients are typically estimated based on the ecosystem’s Net Primary Production (NPP) and the percentage of these elements it contains. The economic value of this service is determined using the market prices of corresponding chemical fertilizers^4^. In this study, this value will be calculated for different land use types in proportion to the coefficients representing the presence of indirect ecological services.

38 $${C}^{n}=\frac{\left[\left({k}_{N}\times {q}_{f}\times {p}_{N}\right)+\left({k}_{f}\times {f}_{P}\times {q}_{f}\times {p}_{P}\right)+\left({k}_{K}\times {q}_{f}\times {p}_{K}\right)\right]\times {\upalpha}_{l}\times A}{Q}$$

#### Total lost value of indirect ecological services

Finally, the total lost value of indirect ecological services calculated in this study includes air purification, oxygen production, water conservation, soil conservation, and nutrient cycling. This total value is computed for each land use type in proportion to the coefficients representing the presence of these services. The final calculation is expressed as a unit cost per ton of extracted ore or rock and is determined using Equation (39).

39 $${C}^{EE}={C}^{a}+{C}^{O}+{C}^{h}+{C}^{s}+{C}^{n}$$

### integration of environmental costs into long-term production planning

To incorporate environmental costs into the long-term production planning of open-pit iron ore mines, it is essential to calculate these costs on a unit basis per ton of rock and integrate them MILP. For this purpose, it must first be determined which types of environmental costs are associated with each type of rock (ore or waste) to enable precise allocation and differentiation of costs.

Regarding the integration of environmental costs into mathematical programming models for long-term production planning in open-pit mines, two different approaches exist. In the first approach, environmental costs are directly incorporated into the objective function of the mathematical model. Given that the objective function in these models is typically defined based on maximizing the NPV of the project, and that this value is calculated based on the economic value of each mining block, environmental costs are added as a cost component to the equation for calculating the economic value of the blocks and, considering the timing of these costs, are reflected in the objective function of the mathematical model. In the second approach, each environmental cost is imposed as a constraint within the mathematical model. For each of these costs and environmental impacts, maximum allowable or permissible values are set according to mining company policies or governmental regulations and are incorporated into the model. Table 3 presents the classification of different environmental costs relevant to long-term production planning in open-pit mines, based on the type of rock (ore or waste).

**Table 3:** Classification of environmental costs based on rock type (ore or waste) *.

**Environmental cost category**	**Subcategory**		**Ore**	**Waste Rock**
Carbon Emissions	Drilling		✓	✓
	Blasting		✓	✓
	Loading		✓	✓
	Hauling		✓	✓
	Ancillary		✓	✓
	Processing (Crushing, Grinding, Separation)		✓	✗
AMD	Acid drainage in waste dumps		✗	✓
Direct Ecological Services	Land acquisition for pit		✓	✗
	Land acquisition for waste dump		✗	✓
	Land acquisition for tailing dam		✓	✗
Land Reclamation Costs	Pit reclamation		✓	✗
	Waste dump reclamation		✗	✓
	Tailing dam reclamation		✓	✗
Indirect Ecological Services	Air purification	Pit	✓	✓
		Waste dump	✗	✓
		Tailing dam	✓	✗
	Oxygen production	Pit	✓	✓
		Waste dump	✗	✓
		Tailing dam	✓	✗
	Water conservation	Pit	✓	✓
		Waste dump	✗	✓
		Tailing dam	✓	✗
	Soil erosion control	Pit	✓	✓
		Waste dump	✗	✓
		Tailing dam	✓	✗
	Nutrient cycling	Pit	✓	✓
		Waste dump	✗	✓
		Tailing dam	✓	✗

*Developed in this study.

### Developed MILP mathematical model

To mathematically model the long-term production planning of open-pit iron ore mines, a MILP model is used. For this purpose, a MILP model is formulated with the objective of maximizing the NPV of the project, while considering the constraints.

Among environmental costs, some cost parameters are block-dependent and vary depending on when and in which year a block is extracted, such as costs associated with carbon emissions from energy consumption during mining and processing operations. On the other hand, certain costs are independent of specific blocks and their extraction timing, with their occurrence predetermined. For example, land acquisition costs and indirect ecological service costs are assumed to be incurred at the beginning of the mine’s life, while land reclamation costs are assumed to be incurred entirely after mine closure. Therefore, these costs are incorporated into the objective function of the model and are discounted according to the timing of their occurrence. In the mathematical model, block-dependent costs are directly included in the calculation of the net economic value of each block, while block-independent costs are added as fixed costs to the objective function and, if necessary, discounted based on their timing Table 4.

40 $$\begin{aligned}NPV\left(X\right)&={\sum}_{i,j,k=1}^{N}{\sum}_{t\in T}\frac{BE{V}_{ijk}}{{\left(1+r\right)}^{t}}\times {X}_{ijk}^{t}\\ & \quad -{\sum}_{i,j,k=1}^{N}\left[{C}_{p}^{d}\times {M}_{ijk}\times {X}_{ijk}^{t}+{C}_{w}^{d}\times {M}_{ijk}^{w}\times {X}_{ijk}^{w}+{C}_{t}^{d}\times {M}_{ijk}^{o}\times {X}_{ijk}^{o}\right]\\ & \quad -\frac{1}{{\left(1+r\right)}^{{T}_{end}}}{\sum}_{i,j,k=1}^{N}\left[\left({M}_{ijk}\times {X}_{ijk}^{t}\right)\times \left({C}_{p}^{r}+{C}_{p}^{EE}\right)+\left({M}_{ijk}^{w}\times {X}_{ijk}^{w}\right)\right.\\ & \quad \left.\times \left({C}_{w}^{r}+{C}_{w}^{EE}\right)+\left({M}_{ijk}^{o}\times {X}_{ijk}^{o}\right) \times \left({C}_{t}^{r}+{C}_{t}^{EE}\right)\right]\end{aligned}$$

41 $$BEV = \left\{ {\begin{array}{*{20}c} {\left( {M_{{ijk}} \times g_{{ijk}} \times Y \times \left( {P - C_{s} } \right)} \right) - \left( {M_{{ijk}} \times \left( {C_{M} + C_{P} + C_{{GHG}}^{o} } \right)} \right)} & {if{\kern 1pt} g{\kern 1pt} {\kern 1pt}> g_{c} } \\ { - \left( {M_{w} \times \left( {C_{M} + C_{{GHG}}^{w} + C_{{AMD}} } \right)} \right)} & {if{\kern 1pt} g{\kern 1pt} {\kern 1pt} < g_{c} } \\ \end{array} } \right.$$

**Table 4:** Demonstration of symbols used in the developed model.

**Symbol**	**Definition**	**Symbol**	**Definition**
$BE{V}_{ijk}$	Block economic value (USD)	r	Annual discount rate (%)
${X}_{ijk}^{t}$	Binary decision variable indicating whether block (i,j,k) is extracted in period t	${M}_{ijk}$	Mass of block (i,j,k) (ton)
${M}_{ijk}^{w}$	Tonnage of block (i,j,k) when classified as waste (ton (	${X}_{ijk}^{w}$	Binary variable; 1 if waste block (i,j,k) is ever extracted, 0 otherwise
${M}_{ijk}^{o}$	Tonnage of block (i,j,k) when classified as ore (ton (	${X}_{ijk}^{o}$	Binary variable; 1 if ore block (i,j,k) is ever extracted, 0 otherwise
${T}_{end}$	Final period of the mine life (years)	$N$	Total number of blocks along each spatial dimension
${g}_{ijk}$	Grade of block (i,j,k( (%)	$Y$	Total metal recovery (%)
P	Metal price (USD/ton)	${C}_{s}$	Selling cost per unit of metal (USD/ton)
${C}_{M}$	Mining cost per block (USD/ton)	${C}_{P}$	Processing cost per block (USD/ton)
${G}_{max}$	Maximum acceptable grade (%)	${G}_{min}$	Minimum acceptable grade (%)
$P{C}_{min}^{t}$	Minimum production capacity in period t (ton)	$P{C}_{max}^{t}$	Maximum production capacity in period t (ton)
${M}_{min}^{t}$	Minimum total mined tonnage in period t (ton)	${M}_{max}^{t}$	Maximum total mined tonnage in period t (ton)

42 $$Y\cdot {X}_{i,j,k}^{t}\le {\sum}_{a=i-1}^{i+1}{\sum}_{b=j-1}^{j+1}{\sum}_{\uptau =1}^{t}{X}_{a,b,k+1}^{\uptau }\hspace{1em}\forall i,j,k,\forall t=\mathrm{1,2},\dots ,T$$

43 $${\sum}_{i,j,k=1}^{N}\left({g}_{ijk}-{G}_{max}\right)\times {M}_{ijk}^{o}\times {X}_{ijk}^{o,t}\le 0\hspace{1em}\forall t=\mathrm{1,2},\dots ,T$$

44 $${\sum}_{i,j,k=1}^{N}\left({g}_{ijk}-{G}_{min}\right)\times {M}_{ijk}^{o}\times {X}_{ijk}^{o,t}\ge 0\hspace{1em}\forall t=\mathrm{1,2},\dots ,T$$

45 $${\sum}_{t=1}^{T}{X}_{ijk}^{t}\le 1\hspace{1em}\hspace{1em}\forall i,j,k=\mathrm{1,2},3,\dots ,N$$

46 $$P{C}_{min}^{t}\le {\sum}_{i,j,k=1}^{N}{M}_{ijk}^{o}\times {X}_{ijk}^{o,t}\le P{C}_{max}^{t}\hspace{1em}\forall t=\mathrm{1,2},\dots ,T$$

47 $${M}_{min}^{t}\le {\sum}_{i,j,k=1}^{N}\left({M}_{ijk}^{o}\times {X}_{ijk}^{o,t}+{M}_{ijk}^{w}\times {X}_{ijk}^{w,t}\right)\le {M}_{max}^{t}\hspace{1em}\forall t=\mathrm{1,2},\dots ,T$$

48 $${X}_{ijk}^{t}={X}_{ijk}^{o,t}+{X}_{ijk}^{w,t}$$

49 $${X}_{ijk}^{o,t}+{X}_{ijk}^{w,t}\le 1$$

Equation (40) represents the objective function of the MILP model, which aims to maximize the NPV of the entire project. The objective function consists of three main components. The first component calculates the present value of each block by considering its extraction time and discounting it to the beginning of the project. The economic value of both ore and waste blocks is determined based on Equation (41). In this study, in addition to extraction and processing costs, time-dependent environmental costs are also incorporated. These costs include GHG emissions and AMD associated with waste materials. The second component of the objective function accounts for direct environmental costs, which are assumed to occur at the beginning of the mine life. These costs include the lost value of direct ecological services associated with land occupied by the pit, waste dump, and tailings dam. The third component captures the costs incurred at the end of the mine life. These costs, discounted to the final year of mining, include reclamation costs, as well as the lost value of indirect ecological services resulting from mining activities.

Equation (42) represents the slope constraint, which serves as a pit stability condition. In this study, the constraint is implemented using a fixed 3×3 precedence, corresponding to a designed slope of 45 degrees, thereby ensuring the geotechnical stability of the pit walls in the case study. According to this constraint, a block can only be mined if the overlying 3×3 set of blocks has already been extracted. Although a 45-degree slope is adopted as the default in this study, in practical applications that involve variable slope requirements or geotechnical domains with different properties, the fundamental principle of defining precedence blocks for each block remains essential. In other words, for each block, the set of overlying blocks that must be extracted first is identified, and this set is then incorporated into the MILP model. Two complementary approaches are proposed to generalize this constraint. First, the precedence set can be defined based on the local allowable slope, so that each block has a customized set enabling extraction under varying slope conditions. Second, geotechnical domains with specific allowable slope angles can be considered, where the precedence set for each block is determined according to the domain it belongs to. Combining these approaches—considering both local slope variations and geotechnical properties—allows the MILP model to realistically capture local slope and rock strength variations, enhancing its applicability to real mines with complex geometries and variable stability conditions. Equations (43) and (44) are defined as grade control constraints to regulate the quality of the plant feed. These constraints ensure that the average grade of the extracted ore in each time period remains within the specified upper and lower bounds. This condition helps maintain uniformity in the plant feed and prevents large fluctuations in product quality. Constraint (45) ensures that each block is extracted only once and within a single time period. In other words, repeated extraction of the same block is not allowed. Constraints (46) and (47) represent the mining and processing capacity limits. These constraints control the total extraction amount (including both ore and waste) as well as the tonnage of ore sent to the processing plant in each time period, ensuring that they remain within the acceptable capacity range. This guarantees the alignment of the production schedule with the operational capacity of both the mine and the processing plant. Constraints (48) and (49) define the binary decision variables. These constraints specify whether each block is allocated as ore or waste, and ensure that a block can only be assigned to one of these two categories in any given time period.

## Model Implementation

**Fig. 5:**
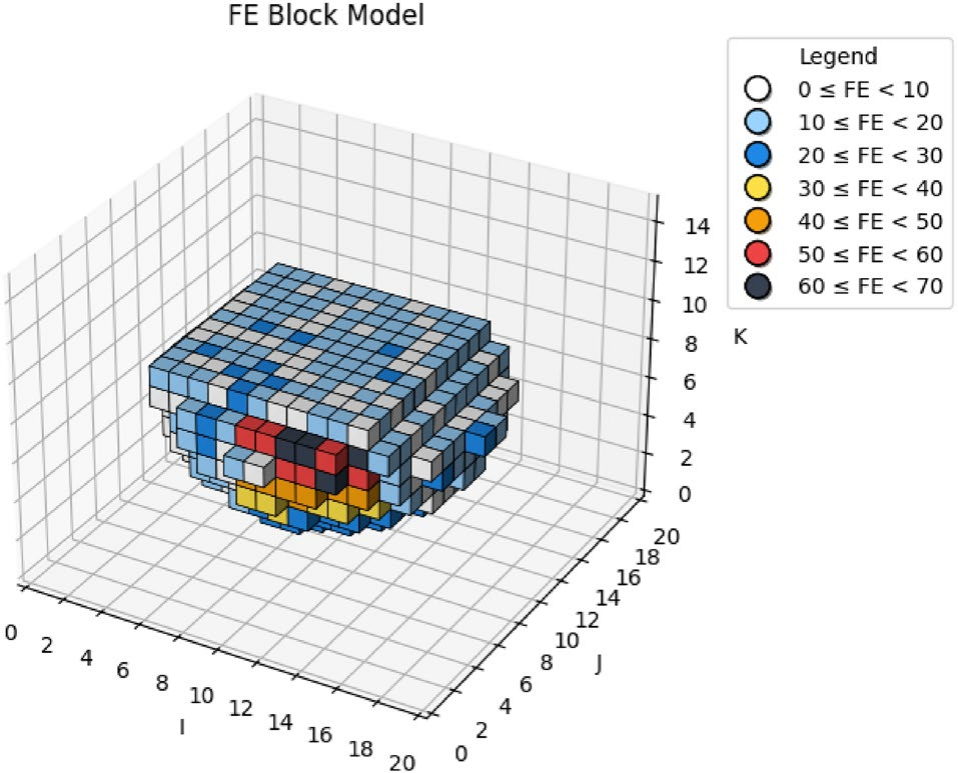
Block model of the hypothetical case study.

In this section, the proposed framework for incorporating environmental costs into long-term production planning of open-pit mines is implemented on a hypothetical iron ore deposit. For this purpose, a block model with the characteristics presented in Table 5 and Fig. 5 was designed, and based on this model, production planning was carried out for a five-year period. The proposed algorithm not only generates the long-term production schedule but also simultaneously determines the ultimate pit limit with the objective of maximizing the NPV. In this case study, environmental costs were explicitly integrated into the objective function of the mathematical model, and the optimization process was carried out with the aim of maximizing the total NPV. The developed mathematical model was implemented in Python (version 3.10.0) using the docplex library (version 2.23.221). Computations were performed on a personal computer with Windows 10 (64-bit), an Intel Core i7 processor, and 16 GB of RAM. The IBM ILOG CPLEX solver was employed due to its high efficiency and proven capability in solving large-scale integer and MILP problems Table 6.

**Table 5:** Specifications of the hypothetical iron ore block model**.**

**Title**	**Unit**	**Value**
Total number of blocks	Number	939
Total ore quantity	Ton	810,000
Total waste quantity	Ton	2,007,000
Average ore grade	%	20
Max grade	%	70
Min grade	%	5
Number of planning periods (mine life)	Year	5

**Table 6:** Economic parameter values considered in long-term production planning.

**Title**	**Unit**	**Value**
Product	-	Iron ore concentrate
Product price	USD/ton	120
Mining cost	USD/ton	1.5
Processing cost	USD/ton	6
Recovery	%	80
Annual discount rate	%	15
Ore and waste density	ton/m^3^	3
Cut-off grade	%	20

To calculate the environmental costs per ton of rock (ore and waste) in the block model, the following assumptions and restrictions were considered:

- The mine under study is located in a desert area.
- Diesel fuel is used during drilling, loading, and hauling operations, while electricity is consumed in crushing, grinding, and magnetic separation processes.
- ANFO explosives are used in blasting, and the reaction is assumed to be complete.
- AMD generation is considered only for sulfide materials in the waste dump.
- Given the desert conditions, the lost value of indirect ecological services is assumed to be zero.
- Land reclamation costs at the end of the mine life and land acquisition costs at the beginning of the mine life are considered.
- Block dimensions are assumed to be 10×10×10 meters, and the rock density (ore and waste) is 3 ton/m^3^.

To evaluate the effect of environmental costs on production scheduling and the ultimate pit limit, three scenarios were designed:

- **Scenario 1:** Long-term production scheduling without considering any environmental costs.
- **Scenario 2:** Long-term production scheduling with the calculation and direct integration of all environmental costs into the mathematical model. In this scenario, all the above-mentioned costs were quantified and incorporated into the model.
- **Scenario 3:** Assessment of the realized impact of environmental costs on the production schedule obtained from Scenario 1. Here, the environmental costs were applied to the schedule of Scenario 1 to calculate the adjusted NPV (ANPV), showing how the omission of environmental costs in the initial planning affects the actual economic performance.

This approach allows a clear comparison between schedules that internalize environmental costs directly in the optimization (Scenario 2) and schedules where environmental costs are applied afterward (Scenario 3), highlighting the importance of including environmental costs in long-term mine planning.

## Results and discussion

In this study, a quantification approach was developed to examine the effect of incorporating environmental costs into long-term production planning of open-pit mines. For comparison purposes, two different scenarios were applied to a hypothetical block model of an iron ore deposit. In the first scenario, production scheduling, extraction sequencing, and ultimate pit limit determination were carried out solely with the objective of maximizing the NPV, considering only mining, processing, and selling costs, while environmental costs were ignored. In contrast, in the second scenario, environmental costs were quantified and integrated into the mathematical model, and production planning was performed based on maximizing the Adjusted Net Present Value (ANPV). This comparison allows for the analysis of the direct impact of incorporating environmental costs on block extraction sequencing, ultimate pit limit, and extractable reserves.

### Production scheduling results

**Fig. 6:**
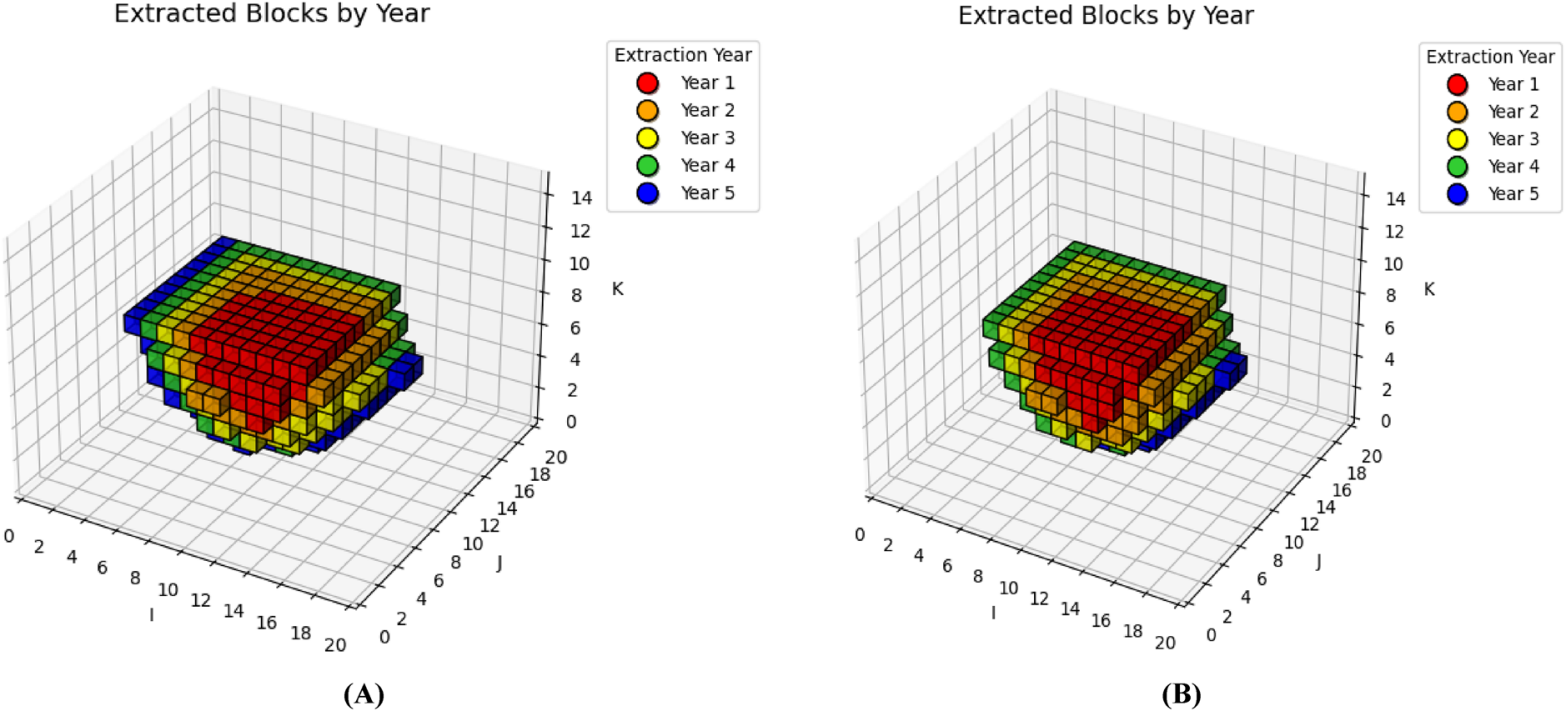
Extraction sequence – (**A**) Scenario 1 without considering environmental costs; (**B**) Scenario 2 with environmental costs incorporated.

In this study, long‑term production planning for an open‑pit mine was carried out under three distinct scenarios. Scenario 1 represents the conventional approach in which environmental costs are not considered, and the objective is solely to maximize the project’s Net Present Value (NPV). In Scenario 2, environmental costs are explicitly incorporated into the objective function of the production planning model; in this case, all relevant environmental cost components described earlier are quantified and integrated into the optimization framework. Scenario 3 evaluates the actual impact of environmental costs on the production schedule derived from Scenario 1. In this scenario, environmental costs are imposed on the Schedule of Scenario 1 to compute the Adjusted Net Present Value, thereby illustrating how neglecting environmental costs during the initial planning stage can influence the project’s true economic performance. As shown in Fig. 6, the ultimate pit limit and resulting production schedule in Scenario 1 are larger than those obtained in Scenario 2. This outcome is expected, as the inclusion of environmental costs increases the overall cost structure and reduces the economic value of certain blocks, rendering their extraction uneconomic. Finally, the comparison of NPV values reported in Table 7 across all scenarios clearly demonstrates the tangible effect of environmental considerations on the project’s profitability.

**Table 7:** Comparison of Net Present Value (NPV) under different production planning scenarios.

**Scenario**	**Production planning setting**	**NPV (USD)**
1 (Scenario 1)	Long-term production planning without environmental costs	14,872,640
2 (Scenario 2)	Long-term production planning with environmental costs included in the objective function	13,669,025
3 (Scenario 1 + Env. Cost Applied)	Environmental costs applied to the ultimate pit limit and production schedule obtained from Scenario 1	11,899,025

A key finding of this study indicates that directly incorporating environmental costs into the objective function of the proposed model increases the total mine NPV by approximately 14.87% compared with conventional approaches in which environmental costs are neglected. This result suggests that integrating environmental costs at the optimization stage not only leads to more realistic decision-making, but also improves the overall economic performance of the project relative to traditional planning approaches. Moreover, explicitly accounting for environmental costs in the long-term production planning process contributes to mitigating environmental impacts while producing mining plans that are more consistent with actual operating conditions. Consequently, the production schedule and ultimate pit limit obtained under Scenario 2 can be regarded as a more realistic and economically optimal basis for decision-making.

**Fig. 7:**
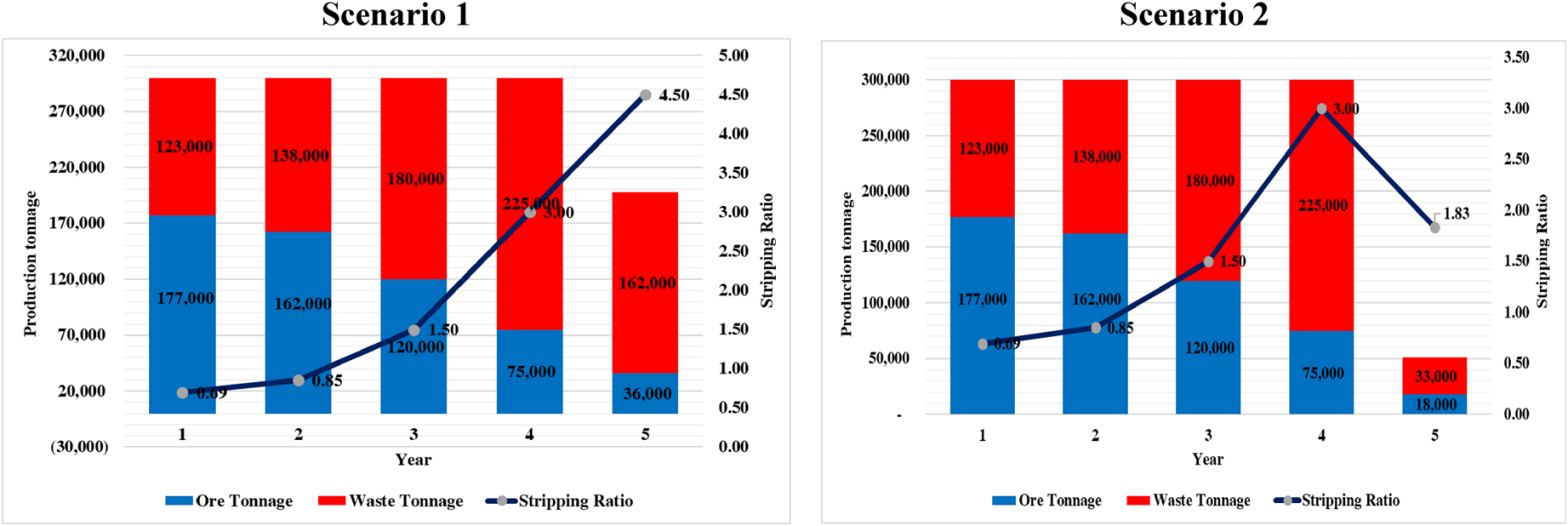
Comparison of annual ore and waste production, and stripping ratio under the two scenarios.

These results are consistent with previous studies that incorporated carbon emission costs into production planning. Similar findings were reported in those studies, where the inclusion of GHG emission costs led to a decrease in the project’s economic indicators, including NPV and extractable reserves^67^^,^^75^^,^^76^. This alignment indicates that the findings of the present model are not only theoretically sound but also consistent with empirical evidence and similar studies in the literature. Although integrating environmental costs into long-term production planning of open-pit mines reduces the NPV and the volume of extractable reserves, it brings the model results closer to real-world conditions. Under such circumstances, the economic assessments of the project provide a more realistic picture and can serve Fig. 7 as an effective tool for decision-making by mine managers and policymakers Table 8.

**Table 8:** Comparison of production outputs and economic indicators between the two scenarios.

**Scenario**	**Parameter**	**Periods**					**Total/Mean** **Value**
		**1**	**2**	**3**	**4**	**5**	
Scenario 1 (Maximize NPV)	Ore Extracted (ton)	177,000	162,000	120,000	75,000	36,000	**570,000**
	Waste Extracted (ton)	123,000	138,000	180,000	225,000	162,000	**828,000**
	Stripping Ratio	0.69	0.85	1.5	3.00	4.50	**2.11**
	Cash Flow ($)	8,146,877	6,341,892	3,054,524	1,409,488	359,509	**19,312,290**
	NPV ($)	7,084,241	4,795,381	2,008,399	805,879	178,740	**14,872,640**
Scenario 2 )Maximize adjusted NPV(	Ore Extracted (ton)	177,000	162,000	120,000	75,000	18,000	**552,000**
	Waste Extracted (ton)	123,000	138,000	180,000	225,000	33,000	**699,000**
	Stripping Ratio	0.69	0.85	1.50	3.00	1.83	**1.58**
	Cash Flow ($)	7,919,665	5,940,278	2,670,440	800,184	155,459	**17,486,026**
	NPV ($)	6,886,665	4,491,704	1,755,858	457,508	77,290	**13,669,025**
Scenario 3 (Scenario 1 + Env. Cost Applied)	Ore Extracted (ton)	177,000	162,000	120,000	75,000	36,000	**570,000**
	Waste Extracted (ton)	123,000	138,000	180,000	225,000	162,000	**828,000**
	Stripping Ratio	0.69	0.85	1.50	3.00	4.50	**2.11**
	Cash Flow ($)	7,919,665	5,940,278	2,670,440	800,184	-3,404,644	**13,925,923**
	NPV ($)	6,886,665	4,491,704	1,755,858	457,508	-1,692,710	**11,899,025**

**Fig. 8:**
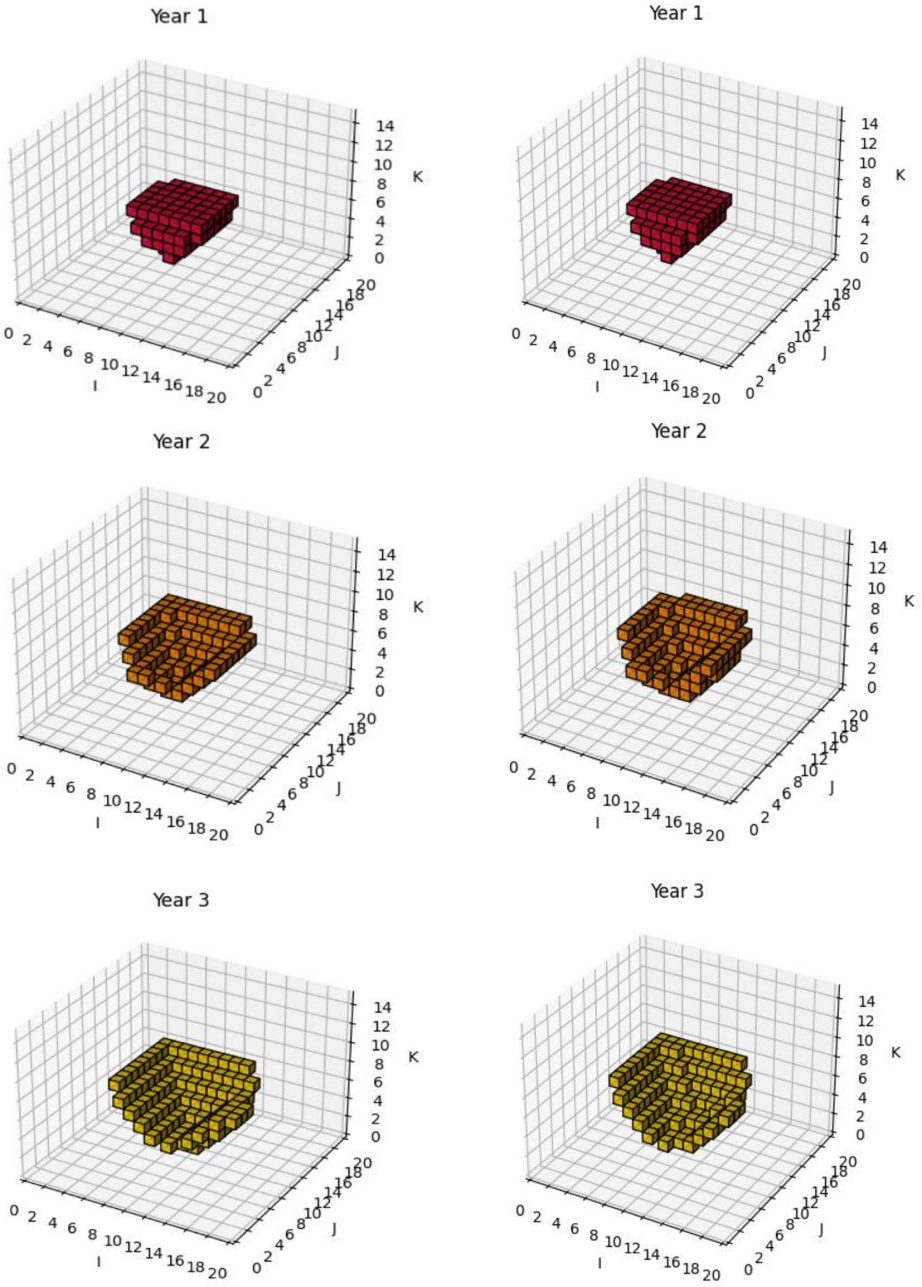

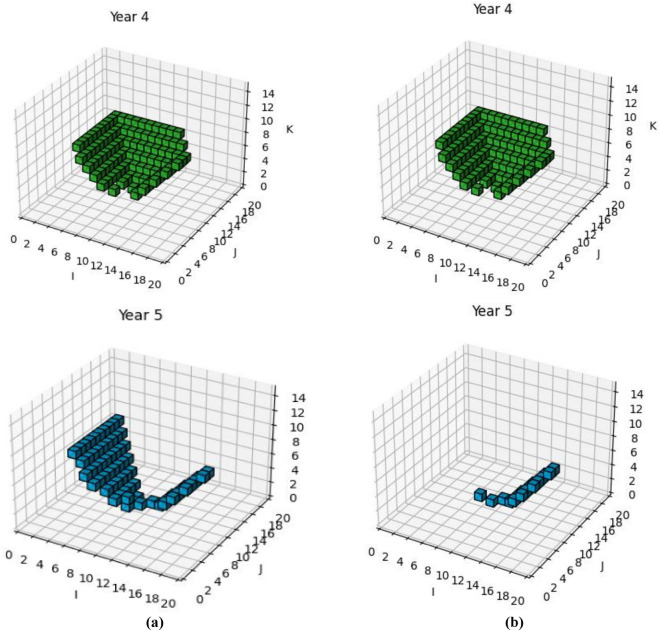
Extracted blocks by year: (**a**) Scenario 1; (**b**) Scenario 2.

In Figure 8, the block extraction sequence over a five-year period is illustrated, broken down by year. As the results indicate, the extraction sequence of high-grade blocks in the first and second years is largely similar in both scenarios. This is because, in the early years, mining operations typically prioritize the extraction of high-grade blocks in order to maximize the overall NPV of the project. Although the inclusion of environmental costs reduces the economic value of these blocks, their relatively high grades and substantial profitability keep them prioritized over lower-grade blocks. However, in the later years, particularly in the fifth years, the extraction sequence differs significantly between the two scenarios, with the number of extracted blocks in Scenario 2 being noticeably lower compared to Scenario 1.

### Cost distribution

In this section, the share of each cost component in the open-pit iron ore production planning is analyzed. For this purpose, the environmental costs incorporated into the model were first calculated per ton of rock (including both ore and waste) and are presented in Table 9. This comparison allows the actual contribution of environmental costs in mining economic decision-making to be identified, thereby highlighting the scientific value of the proposed model. It should be noted that the environmental costs reported in Table 9 were calculated prior to the implementation of the production scheduling model, based on the formulations developed in the methodology section of this study. The results emphasize that environmental costs may represent a significant portion of the total expenditures, indicating their potential to substantially influence production planning strategies and overall project economics.

**Table 9:** Calculated environmental costs for the case study**.**

**Cost type**	**Value ($/ton)**	
	**Ore**	**Waste**
C_GHG_	0.314	0.314
C_AMD_	0	0 – 30*
C^d^_p_	0.00067	0.00067
C^d^_w_	0	0.00098
C^d^_t_	0.00363	0
C^r^_p_	0.00667	0.00667
C^r^_w_	0	0.010
C^r^_t_	0.0363	0
C^EE^	0.0000032	0.00000125

*It varies depending on the acid generation potential.

**Fig. 9:**
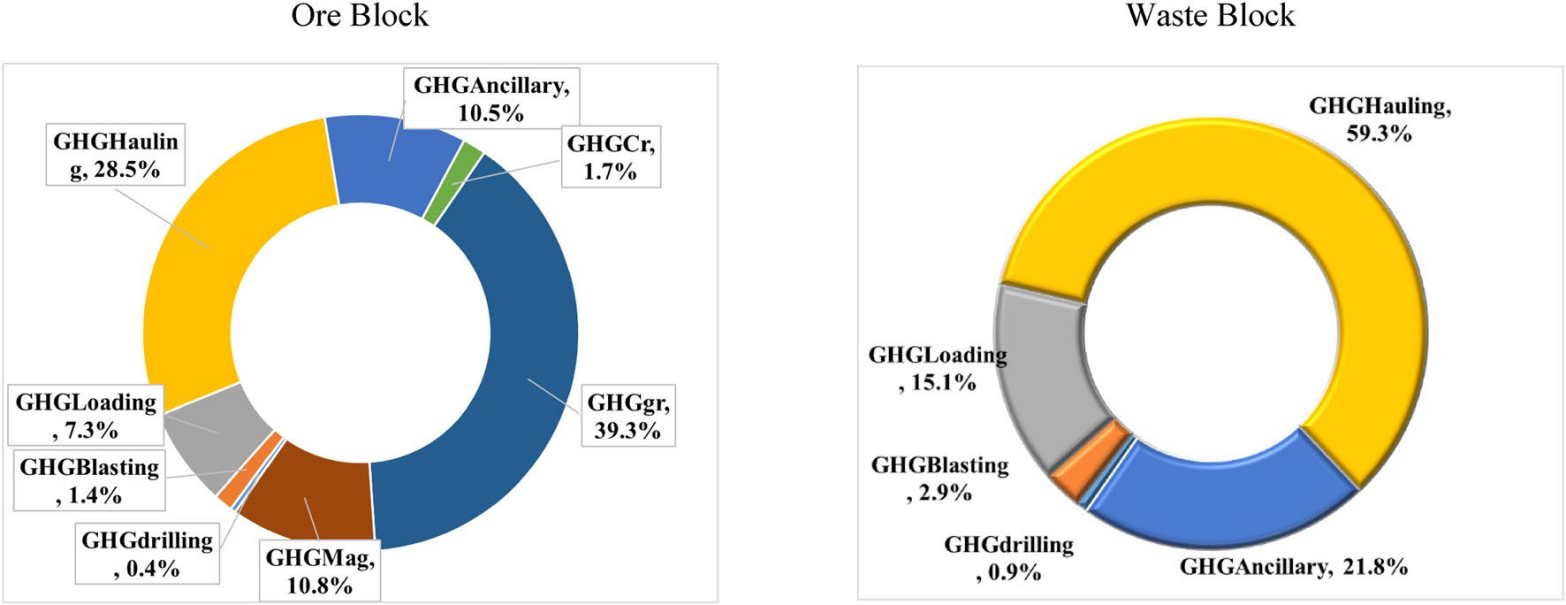
Contribution of each activity to carbon emission**.**

In Figure 9, the contribution of different mining and processing operations to GHG emissions from iron ore production is illustrated. According to the results, 5 main operations—grinding (processing), hauling and transportation (mining), magnetic separation (processing), and support activities (both mining and processing), along with loading (mining)—account for the largest shares of emissions, with contributions of 39.3%, 28.5%, 10.8%, 10.5%, and 7.3%, respectively. These findings are consistent with the study by Mirzehi and Afrapoli^67^, in which only mining operations were considered, and material hauling, support activities, and loading were identified as the primary sources of emissions. This consistency highlights the validity of the developed model in the present study and its potential applicability to other iron ore mining operations.

After implementing the production scheduling under the two defined scenarios, the share of each cost component in the total extraction of the recoverable reserve was examined and compared. The results of this comparison are presented in Table 10, where the relative contribution of each cost category is shown after the complete extraction of the recoverable reserve in each scenario.

**Table 10:** Comparative analysis of cost shares between Scenario 1 and Scenario 2.

**Cost Type**	**Scenario 1**			**Scenario 2**	
	**Value ($)**	**Share (%)**		**Value ($)**	**Share (%)**
Processing	3,420,000	62.00%		3,312,000	47.97%
Mining	2,097,000	38.00%		1,876,500	27.18%
GHG Emissions	0	0.00%		597,231	8.65%
AMD	0	0.00%		1,080,000	15.64%
Reclamation (Tailing)	0	0.00%		20,038	0.29%
Reclamation (Pit)	0	0.00%		8,340	0.12%
Reclamation (Dump)	0	0.00%		6,480	0.09%
Land (Tailing)	0	0.00%		2,004	0.03%
Land (Pit)	0	0.00%		834	0.01%
Land (Dump)	0	0.00%		635	0.01%
Env Services (Pit)	0	0.00%		0	0.00%
Env Services (Dump)	0	0.00%		0	0.00%
Env Services (Tailing)	0	0.00%		0	0.00%
Summation	5,517,500	100%		6,904,062	100%

**Fig. 10:**
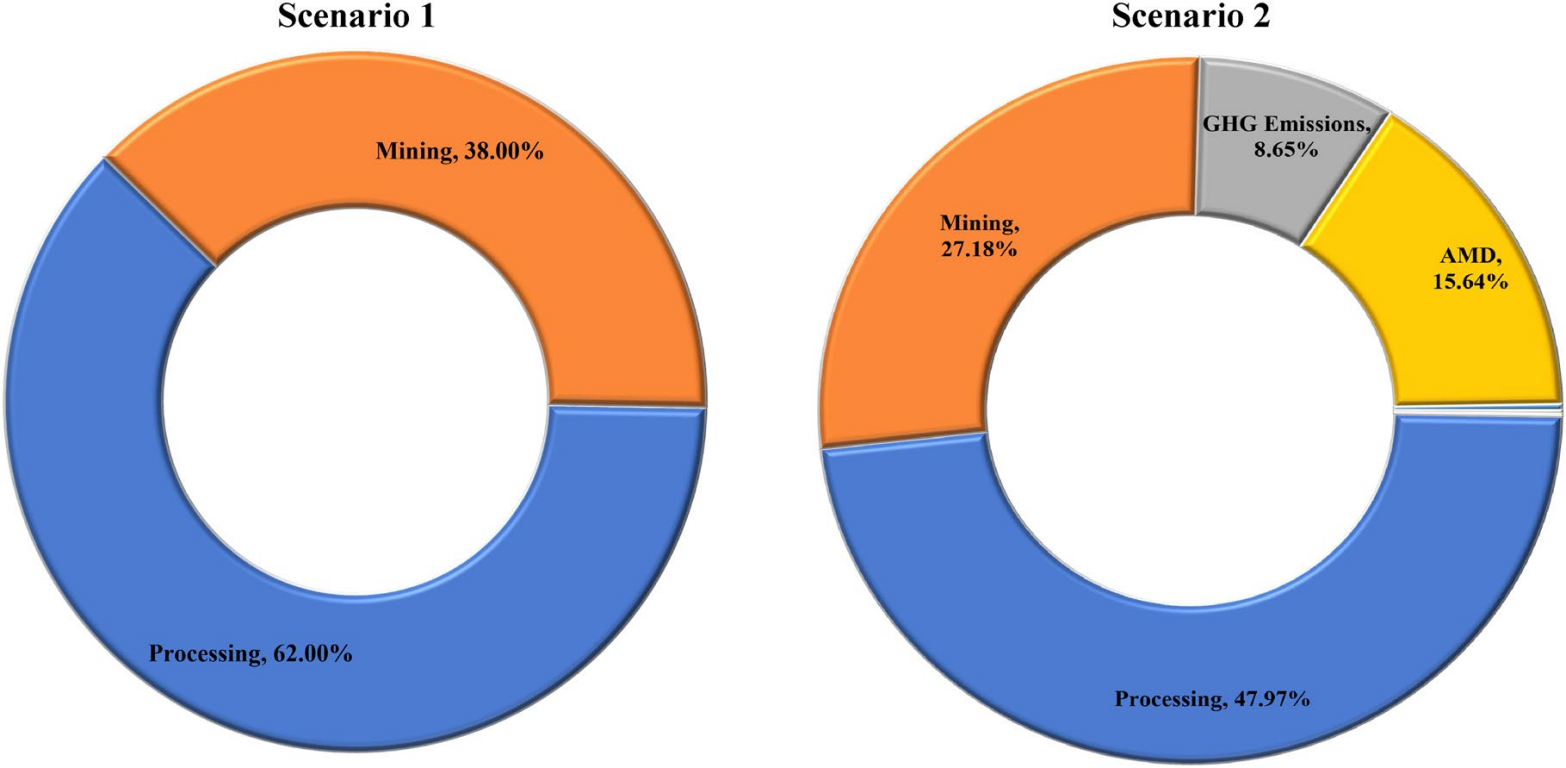
Comparative analysis of cost shares between Scenario 1 and Scenario 2.

Given that the processing cost per ton of ore is assumed to be more than twice the mining cost, and considering the total volumes of ore and waste extracted over the mine life, the total processing cost accounts for the largest share of the combined mining costs in this case study. Following mining and processing costs, as expected, GHG emission costs and AMD control costs, along with the costs of tailings dam reclamation, represent the next largest shares of the total costs for the iron ore mine under investigation. The results indicate that, overall, more than 15% of the total project costs in this case study are attributed to environmental costs. This highlights the significant role and importance of incorporating environmental costs into production planning processes, and demonstrates that ignoring these components may lead to unrealistic cost estimations and, consequently, flawed managerial decision-making Fig. 10.

### Discussion

In this study, for the first time, all environmental costs influencing long-term production planning in open-pit mines were identified and classified into five main categories. To quantify these costs, specific formulations and equations were developed. Furthermore, a new MILP model for long-term production planning was developed, in which the quantified environmental costs were incorporated into the objective function alongside conventional mining and processing costs. Finally, the proposed model was implemented on a hypothetical iron ore mine as a case study.

The results of this study clearly demonstrate that incorporating environmental costs into long-term production planning for open-pit mines has a direct and substantial impact on both the economic and technical performance of the project. A comparison of the two investigated scenarios shows that, although Scenario 1—where environmental costs are ignored—yields larger recoverable reserves, this apparent improvement does not reflect the project’s true economic performance. The results indicate that explicitly integrating environmental costs into the objective function of the proposed model leads to an approximately 14.87% increase in net present value compared with the scenario 3 (conventional scenario in which environmental costs are not considered for mine planning and explicitly integrating environmental costs into the objective function for NPV). This outcome suggests that several blocks classified as profitable under traditional production planning assumptions become uneconomic once environmental costs are properly accounted for. Consequently, the results obtained from Scenario 2 provide a more realistic representation of the project’s economic conditions and lead to a production plan that is closer to the true optimal solution. The extraction sequence analysis further reveals that, while high-grade blocks remain a priority in the early years of mine life, a clear divergence emerges in the later years, with fewer blocks extracted under Scenario 2 than under Scenario 1. This finding confirms that environmental costs influence not only the economic value of the project but also operational extraction strategies. Moreover, the cost distribution analysis shows that processing accounts for the largest share of total expenditures, followed by costs associated with greenhouse gas emissions, acid mine drainage control, and tailings dam reclamation. Collectively, these environmental costs represent nearly 25% of total project expenditures in the present case study. These findings highlight the critical importance of incorporating environmental costs into the economic evaluation of mining projects and suggest that neglecting them may result in unrealistic cost estimates, suboptimal managerial decision-making, and undesirable outcomes.

Finally, comparing the results of this study with previous research that incorporated greenhouse gas emission costs into production planning models demonstrates the consistency of the findings. This consistency not only confirms the validity of the developed model but also underscores its potential generalizability to other iron ore mining operations. Accordingly, it can be concluded that integrating environmental costs into production optimization models represents a scientifically robust and credible approach, while also serving as a practical tool for informed decision-making by mine managers and policymakers.

## Conclusion

Based on the principles of sustainable mining, mineral extraction should be carried out in a manner that satisfies current societal demands while maintaining an appropriate balance with the surrounding environment and ecosystems. Achieving such a balance requires that environmental considerations be systematically integrated into technical and economic decision-making processes in mining, particularly in long-term production planning. In this study, for the first time, all environmental costs affecting long-term production planning in open-pit mines were comprehensively identified and classified into five main categories. To quantify these costs, novel formulations and methodologies were developed, enabling their calculation on a per-ton-of-rock basis. In order to integrate these costs into production planning, environmental costs were further disaggregated according to rock type and timing of expenditure. Subsequently, a new mixed-integer linear programming (MILP) model was developed in which these costs were directly embedded in the objective function of a three-dimensional block model to generate a sustainable long-term production plan. The results indicate that incorporating environmental costs leads to a reduction in extracted tonnage and recoverable reserves relative to the baseline case, as well as changes in the block extraction sequence. However, a more detailed analysis reveals that directly embedding these costs into the objective function of the proposed model results in the project NPV under Scenario 2 being approximately 14.87% higher than that obtained from the Scenario 3 (conventional scenario in which environmental costs are not considered for mine planning and explicitly integrating environmental costs into the objective function for NPV). This finding demonstrates that production planning based on Scenario 1 exhibits weaker economic performance once all relevant costs are fully accounted for, compared with the solution derived from the proposed framework. Accordingly, the ultimate pit limit and production schedule obtained under Scenario 2, which are developed based on the proposed framework of this study, not only reduce the environmental impacts associated with mining activities but also provide a more realistic assessment of the project’s economic value, leading to an improvement in the project’s actual NPV relative to the baseline case. In other words, Scenario 2 establishes a more appropriate balance between economic and environmental considerations and offers a more robust and reliable solution for long-term production planning in open-pit mines. Overall, by systematically integrating quantified environmental costs into a three-dimensional mathematical framework, this study advances beyond previous two-dimensional approaches and enables the simultaneous evaluation of economic and environmental aspects. The proposed framework can serve as a practical tool for mine managers and designers to develop more realistic production plans, while also providing policymakers with a quantitative basis for informed decision-making. Future research may further enhance this framework through the development of multi-objective models and the application of heuristic and meta-heuristic algorithms to large-scale problems, thereby improving both computational efficiency and generalizability.

## Electronic supplementary material

Below is the link to the electronic supplementary material.

Supplementary Information.

## Data Availability

The data used/generated in this study are provided in the Supplementary Information. The full hypothetical block model dataset is available as an Excel file. Additional data and information can be provided by the corresponding author upon reasonable request, subject to feasibility.

## Appendix A

See Table 11, Table 12, Table 13.

**Table 11:** Formulations developed in this study for quantifying environmental costs.

**Category**	**Formula / Expression**	**Source**	**Formula Number**
GHG emissions in drilling operations	${GHG}_{Drilling}=\left(\frac{P\times N\times {t}_{hole}\times 3.6}{{\upeta}_{Driller}\times {m}_{B}}\right)\times \zeta$	Developed in this study	**3**
	${GHG}_{drillig}={R}_{d}\times \frac{{M}_{d}\times BF}{{P}_{r}}\times \delta$		**4**
GHG Emissions in blasting operations	$GH{G}_{Blast}=LF\times \left(\left({R}_{ANFO}\times {Q}_{A}\right)+\left({R}_{emulsion}\times {Q}_{E}\right)\right)\times {10}^{-3}$	Developed in this study	**6**
GHG Emissions in loading operations	${GHG}_{loading}={D}_{l}\times \delta$	Developed in this study	**8**
GHG Emissions in haulage operations	${GHG}_{Hauling}=\left(\frac{{T}_{cycle}\cdot FCR}{60\cdot {m}_{Truck}\cdot EF}\right)\times \delta$	Developed in this study	**10**
GHG Emissions in crushing	${GHG}_{cr}=3.6\times 10\times {W}^{cr}\times \left(\frac{1}{\sqrt{{P}_{80}^{cr}}}-\frac{1}{\sqrt{{F}_{80}^{cr}}}\right)\times \zeta$	Developed in this study	**13**
GHG Emissions in grinding	${GHG}_{gr}=3.6\times 10\times {W}^{gr}\times \left(\frac{1}{\sqrt{{P}_{80}^{gr}}}-\frac{1}{\sqrt{{F}_{80}^{gr}}}\right)\times \zeta$	Developed in this study	**14**
GHG Emissions in magnetic separation	${GHG}_{Mag}=\frac{{P}_{mag}\times {T}_{mag}\times 3.6}{{m}_{ore}}\times \zeta$	Developed in this study	**15**
Total ore GHG	$$\begin{aligned}{GHG}_{O}&={GHG}_{Drilling}+GH{G}_{Blast}+{GHG}_{loading}+{GHG}_{Hauling}\\ &\quad +{GHG}_{Ancillary}+{GHG}_{cr}+{GHG}_{gr}+{GHG}_{Mag}\end{aligned}$$	Developed in this study	**16**
Total waste GHG	$$\begin{aligned}{GHG}_{W}&={GHG}_{Drilling}+GH{G}_{Blast}+{GHG}_{loading}\\ & \quad +{GHG}_{Hauling}+{GHG}_{Ancillary}\end{aligned}$$	Developed in this study	**17**
Cost of greenhouse gas emissions from the ore	${C}_{GHG}^{o}=\frac{{T}_{GHG}\times {GHG}_{O}}{1000}$	Developed in this study	**19**
Cost of greenhouse gas emissions from the waste	${C}_{GHG}^{w}=\frac{{T}_{GHG}\times {GHG}_{W}}{1000}$	Developed in this study	**18**
Cost of AMD generation	${C}_{AMD}={\gamma}_{i}\times \alpha \times {C}_{base}$	Developed in this study	**24**
Lost value of direct ecological service cost	${A}_{w}=\frac{{Q}_{w}\cdot {f}_{w}}{{\uprho}_{w}\cdot {H}_{w}}\cdot {s}_{w}$	Developed in this study	**26**
	${C}_{w}^{d}=\frac{{A}_{w}\times {c}_{g}}{{Q}_{w}}$	Developed in this study	**27**
	${A}_{t}=\frac{{Q}_{t}}{{\uprho}_{t}\cdot {H}_{t}}\cdot {s}_{t}$	Developed in this study	**28**
	${C}_{t}^{d}=\frac{{A}_{t}\times {c}_{g}}{{Q}_{O}}$	Developed in this study	**29**
	${Q}_{t}=\left(1-\frac{{G}_{feed}\times R}{{G}_{conc}}\right)\times {Q}_{O}$	Developed in this study	**30**

**Table 12:** Formulations adopted from the literature for quantifying environmental costs.

**Category**	**Formula / Expression**	**Source**	**Formula Number**
GHG Emissions in drilling operations	${GHG}_{Drilling}=\left(\frac{A\cdot L\cdot N\cdot {E}_{V}}{{\upeta}_{Driller}\cdot {m}_{B}}\right)\times \zeta$	^66^	**1**
	${GHG}_{Drilling}=\left(\frac{{E}_{V}}{{\upeta}_{Driller}\cdot {\rho}_{rock}}\right)\times \zeta$	^66^	**2**
GHG Emissions in blasting operations	$GH{G}_{Blast}={Q}_{E}\times LF$	^66^	**5**
GHG Emissions in loading operations	${GHG}_{loading}=\left(\frac{{P}_{L}\times T}{{\upeta}_{Loader}\times {m}_{Truck}}\right)\times \zeta$	^66^	**7**
GHG Emissions in haulage operations	${GHG}_{Hauling}=\left(\frac{9.81\times S\times \left({m}_{Truck}\times i\%+\left({R}_{s}\%+{R}_{t}\%\right)\times \left(2\times {M}_{Truck}-{m}_{Truck}\right)\right)}{{m}_{Truck}}\right)\times \zeta$	^66^	**9**
GHG Emissions in ancillary activities	${GHG}_{Ancillary}={D}_{a}\times \delta$	^67^	**12**
Cost of AMD generation	$AM{D}_{Pot}=f\left(NNP,NPR\right)$	^66^	**20**
	$NNP=NP-AP$	^66^	**21**
	$NPR=\frac{NP}{AP}$	^66^	**22**
	$NP = 83.3 \times {g}_{c} ;\hspace{1em}AP = 31.25 \times {g}_{s}$	^66^	**23**
Lost value of direct ecological service cost	${C}_{p}^{d}=\frac{{A}_{p}\times {c}_{g}}{{Q}_{tot}}$	^72^	**25**
Reclamation costs	${C}_{p}^{r}=\frac{{A}_{p}\times {c}_{r}}{{Q}_{tot}}$	^72^	**31**
	${C}_{w}^{r}=\frac{{A}_{w}\times {c}_{r}}{{Q}_{w}}$	^72^	**32**
	${C}_{t}^{r}=\frac{{A}_{t}\times {c}_{r}}{{Q}_{O}}$	^72^	**33**
Air purification value	${C}^{a}=\frac{\left({\sum}_{i=1}^{n}{Q}_{i}\times {C}_{i}\right)\times {\alpha}_{l}\times A}{Q}$	^72^	**34**
Oxygen production value	${C}^{O}=\frac{\left({q}_{f}\times {\lambda}_{o}\times {c}_{o}\times {\alpha}_{l}\right)\times A}{Q}$	^72^	**35**
Water conservation value	${C}^{h}=\frac{\left(10\times j\times k\times {r}_{h}\times {c}_{h}\times {\upalpha}_{l}\right)\times A}{Q}$	^72^	**36**
Soil erosion control value	${C}^{s}=\frac{\left(\frac{{S}_{p}\times\uprho \times h\times v}{\mathrm{10,000}}\times {\upalpha}_{l}\right)\times A}{Q}$	^72^	**37**
Nutrient cycling value	${C}^{n}=\frac{\left[\left({k}_{N}\times {q}_{f}\times {p}_{N}\right)+\left({k}_{f}\times {f}_{P}\times {q}_{f}\times {p}_{P}\right)+\left({k}_{K}\times {q}_{f}\times {p}_{K}\right)\right]\times {\upalpha}_{l}\times A}{Q}$	^72^	**38**

## Appendix B

**Table 13:** Nomenclature full.

**Symbol**	**Description**	**Unit**
${GHG}_{Drilling}$	${CO}_{2eq}$ emissions from drilling	kg ${CO}_{2eq}$/ton
A	Area of the drilling section	cm^2^
${E}_{V}$	Drilling specific energy	MJ/cm^3^
L	Drilling length	Cm
N	Number of drillings per block	-
${\upeta}_{Driller}$	Driller’s efficiency	-
${m}_{B}$	Mass of the block	Ton
${\rho}_{rock}$	Rock density	ton/m^3^
$\zeta$	Carbonization’s coefficient of electrical power	kg ${CO}_{2eq}$/MJ
P	Drilling machine power	kW
N	Number of holes per block	-
${t}_{hole}$	Drilling time per hole	H
${R}_{d}$	The hourly rate of diesel consumption during drilling	l/h
${M}_{d}$	Drill meters needed per ton of mined rock	m/ton
$BF$	The percentage of mined rock that necessitates drilling and blasting	%
${P}_{r}$	The speed at which the drill penetrates	m/h
$\delta$	Carbonization’s coefficient	kg CO₂-eq/l
$GH{G}_{Blast}$	Greenhouse gas emissions from blasting operations	kg CO₂-eq/ton
LF	Specific charge	kg of ANFO/ton
${Q}_{E}$	The amount of CO2e generated from emulsion combustion	kg CO₂-eq/kg emulsion
${GHG}_{loading}$	Greenhouse gas emissions from loading operations	kg CO₂-eq/ton
${P}_{L}$	Loader power	MW
$T$	Loading time	S
${\upeta}_{Loader}$	Loader efficiency	-
${m}_{Truck}$	Truck capacity	Ton
${D}_{l}$	Diesel consumption rate for loading operations	l/ton
${GHG}_{Hauling}$	CO₂-equivalent emissions from haulage operation	kg CO₂-eq/ton
$S$	Haul distance	M
$i$	Road gradient	%
${R}_{s}$	Road surface rolling resistance	%
${R}_{t}$	Internal resistance of the truck	%
${M}_{Truck}$	Empty truck weight	Ton
${T}_{cycle}$	Total haulage cycle time	Min
$FCR$	Fuel consumption rate	L/h
$EF$	Truck efficiency factor	-
$D$	One-way haul distance	Km
${V}_{loaded}$	Average truck speed when loaded	km/h
${V}_{empty}$	Average truck speed when empty	km/h
${T}_{load}$	Loading time	Min
${T}_{dump}$	Dumping time	Min
${T}_{wait}$	Waiting time	Min
${GHG}_{Ancillary}$	CO₂-equivalent emissions from ancillary activities	kg CO₂-eq/ton
${D}_{a}$	Diesel consumption rate in ancillary activities	l/ton
${GHG}_{cr}$	CO₂-eq emissions associated with Crushing	kg CO₂-eq/ton
${W}^{cr}$	Bond Crushing Work Index	kWh/ton
${P}_{80}^{cr}$	80% passing size of grinding product	µm
${F}_{80}^{cr}$	80% passing size of grinding feed	µm
${GHG}_{gr}$	CO₂-eq emissions associated with Grinding	kg CO₂-eq/ton
${W}^{gr}$	Bond Grinding Work Index	kWh/ton
${P}_{80}^{gr}$	80% passing size of grinding product	µm
${F}_{80}^{gr}$	80% passing size of grinding feed	µm
${GHG}_{Mag}$	Greenhouse gas emissions from magnetic separation	kg CO₂-eq/ton
${P}_{mag}$	Power of magnetic separator	kW
${T}_{mag}$	Operating time of magnetic separation process	H
${m}_{ore}$	Mass of processed ore	Ton
${C}_{GHG}^{w}$	Carbon cost associated with waste rock	USD/ton
${T}_{GHG}$	Carbon tax	USD/ton CO₂-eq
${GHG}_{W}$	CO₂-equivalent emissions per ton of waste rock	kg CO₂-eq/ton
${C}_{GHG}^{o}$	Carbon cost associated with ore	USD/ton
${GHG}_{O}$	CO₂-equivalent emissions per ton of ore	kg CO₂-eq/ton
$AM{D}_{Pot}$	Acid Mine Drainage potential	-
$NNP$	Net Neutralization Potential	kg CaCO₃/ton
NPR	Neutralization Potential Ratio	-
NP	Neutralizing Potential	kg CaCO₃/ton
AP	Acidic Potential	kg CaCO₃/ton
${\mathrm{g}}_{\mathrm{c}}$	Percentage of carbonate content	%
${\mathrm{g}}_{\mathrm{s}}$	Total sulfur content (primarily pyrite)	%
${C}_{AMD}$	AMD control cost	USD/ton
${\gamma}_{i}$	AMD intensity coefficient based on risk level	-
$\alpha$	Regional/climatic adjustment factor	-
${C}_{base}$	Base AMD control cost under standard conditions	USD/ton
${C}_{p}^{\mathrm{d}}$	Land disturbance cost of mined rock	USD/ton
${A}_{p}$	Area of land disturbed in the maximum resource pit	Ha
${c}_{g}$	Unit price or direct ecological value per unit area	USD/ha
${Q}_{tot}$	Total rock extracted from the maximum resource pit	Ton
${A}_{w}$	Waste dump area	Ha
${Q}_{w}$	Total waste from the maximum resource pit	Ton
${f}_{w}$	Swell factor of waste rock	-
${\uprho}_{w}$	Density of waste rock	ton/m^3^
${H}_{w}$	Height of waste dump	M
${s}_{w}$	Waste dump shape factor	-
${C}_{w}^{\mathrm{d}}$	Land disturbance cost (direct ecological service cost) per ton of waste	USD/ton
${A}_{t}$	Tailings dam area	Ha
${Q}_{t}$	Mass of tailings	Ton
${\uprho}_{t}$	Density of tailings	ton/m^3^
${H}_{t}$	Height of tailings dam	M
${s}_{t}$	Tailings dam shape factor	-
${C}_{t}^{\mathrm{d}}$	Tailings dam Land disturbance cost (direct ecological service cost)	USD/ton
${\mathrm{Q}}_{O}$	Mass of Ore	Ton
${G}_{feed}$	Grade of plant feed	%
$R$	Processing recovery	%
${G}_{conc}$	Grade of final concentrate	%
${C}_{p}^{r}$	Reclamation cost of pit-disturbed land	USD/ton
${c}_{r}$	Unit reclamation cost	USD/ha
${C}_{w}^{r}$	Reclamation cost of waste dump land	USD/ton
${C}_{t}^{r}$	Reclamation cost of tailings dam land	USD/ton
${\mathrm{C}}^{\mathrm{a}}$	Unit cost of indirect ecological services loss	USD/ton
${Q}_{i}$	Quantity of pollutant i removed or mitigated by the ecosystem	kg/ha/year
${C}_{i}$	Cost of removing or replacing pollutant i using artificial methods	USD/kg
${\alpha}_{l}$	Weighting coefficient based on land type	-
${C}^{O}$	Unit cost of oxygen production services	USD/ton
${q}_{f}$	Net Primary Production (NPP) of the ecosystem	ton/ha/year
${c}_{o}$	Industrial cost of oxygen production	USD/ton
${C}^{h}$	Unit cost of water conservation services	USD/ton
$j$	Mean annual precipitation	mm/year
$k$	Ratio of effective rainfall contributing to runoff	-
${r}_{h}$	Runoff reduction coefficient	-
${c}_{h}$	Unit cost of water storage	USD/m^3^
${C}^{s}$	Unit cost of soil conservation services	USD/ton
${S}_{p}$	Soil retention capacity of the ecosystem	ton/ha/year
$\rho$	Soil bulk density	ton/m^3^
$h$	Thickness of soil layer preserved	M
$v$	Annual income generated by the conserved land	USD/ha/year
${C}^{n}$	Unit cost of soil conservation services	USD/ton
${k}_{N}$	Nitrogen content percentage in NPP	%
${q}_{f}$	Net Primary Production (NPP) of the ecosystem	ton/ha^2^/year
${p}_{N}$	Market price of nitrogen fertilizer	USD/ton
${k}_{f}$	Phosphorus content percentage in NPP	%
${f}_{P}$	Conversion factor from P to P₂O₅	-
${p}_{P}$	Market price of phosphorus fertilizer	USD/ton
${k}_{K}$	Potassium content percentage in NPP	%
${p}_{K}$	Market price of potassium fertilizer	USD/ton
${C}^{EE}$	Total Lost Value of Indirect Ecological Services	USD/ton
